# Temporal trends in the incidence of malignant and nonmalignant primary brain and central nervous system tumors by the method of diagnosis in England, 1993–2017

**DOI:** 10.1093/neuonc/noad001

**Published:** 2023-01-04

**Authors:** Usama M. Ali, Diana R. Withrow, Andrew D. Judge, Puneet Plaha, Sarah C. Darby

**Affiliations:** Nuffield Department of Population Health, University of Oxford, Oxford, UK; Nuffield Department of Primary Care Health Sciences, University of Oxford, Oxford, UK; Nuffield Department of Orthopaedics, Rheumatology, and Musculoskeletal Sciences, University of Oxford, Oxford, UK; Bristol NIHR Biomedical Research Centre and University of Bristol, Bristol, UK; Nuffield Department of Surgical Sciences, University of Oxford, Oxford, UK; Department of Neurosurgery, John Radcliffe Hospital, Oxford University Hospital NHS Foundation Trust, Oxford, Oxford, UK; Nuffield Department of Population Health, University of Oxford, Oxford, UK

**Keywords:** brain tumor, cancer registry, diagnosis, epidemiology, incidence

## Abstract

**Background:**

Several studies report increases in the incidences of primary central nervous system (CNS) tumors. The reasons for this are unclear.

**Methods:**

Data on all 188 340 individuals diagnosed with a primary CNS tumor in England (1993–2017) were obtained from the National Cancer Registration and Analysis Service. Data on all computerized tomography (CT) head and magnetic resonance imaging (MRI) brain scans in England (2013–2017) were obtained from the National Health Service Digital. Age-sex-standardized annual incidence rates per 100 000 population (ASR) were calculated by calendar year, tumor behavior, tumor location, and method of diagnosis. Temporal trends were quantified using average annual percent change (AAPC).

**Results:**

The ASR for all CNS tumors increased from 13.0 in 1993 to 18.6 in 2017 (AAPC: +1.5%, 95% CI: 1.3, 1.7). The ASR for malignant tumors (52% overall) remained stable (AAPC: +0.5%, 95% CI: −0.2, 1.3), while benign tumors (37% overall) increased (AAPC: +2.6%, 95% CI: 1.2, 4.0). Among the 66% of benign tumors that were microscopically confirmed, the ASR increased modestly (AAPC: +1.3%, 95% CI: 0.5, 2.1). However, among the 25% of benign tumors that were radiographically confirmed, the ASR increased substantially (AAPC: 10.2%, 95% CI: 7.9, 12.5), principally driven by large increases in those who are aged 65+ years. The rate of CT head scans in Accident & Emergency (A&E) increased during 2013–2017, with especially large increases in 65–84 and 85+-year-olds (AAPCs: +18.4% and +22.5%).

**Conclusions:**

Increases in CNS tumor incidence in England are largely attributable to the greater detection of benign tumors. This could be the result of the increasing use of neuroimaging, particularly CT head scans in A&E in people who are aged 65+ years.

## Introduction

Primary tumors of the brain and central nervous system (CNS), hereafter collectively referred to as CNS tumors, comprise a heterogeneous group of neoplasms originating in or impinging on the brain, spinal cord, meninges (3-layered membrane that covers the brain and spinal cord), or the endocrine glands located at the base of the brain.^1^ CNS tumors can be categorized as malignant or nonmalignant (ie, exhibiting benign or uncertain behavior). While malignant tumors are usually associated with poor prognosis, nonmalignant tumors can also result in adverse outcomes through raised intracranial pressure, cerebral edema, and compression of healthy tissue. These can lead to significant neurological deficits and in the most severe cases can result in fatality. Over 90% of primary CNS tumors occur in and around the brain while spinal cord tumors account for the remainder.^2^

The morbidity and mortality caused by CNS tumors are disproportionate to their incidence.^3^ Malignant brain tumors were the leading cause of cancer death in people aged under 40 years in England in 2018.^4^ Many survivors experience considerable morbidity, due to either the tumor itself or the side effects of treatment, causing the reduction in, or loss of independent functioning and long-term neurological deficits.^5^ Globally, an increase has been reported in the burden of CNS tumors over the past 3 decades, with increasing incidence across all sociodemographic levels and geographic regions except for eastern Europe, where it has remained stable.^6^

Data on the incidence rates of CNS tumors in England comes mainly from two studies, one that found an increase during 1979–1992 followed by a leveling off during 1993 to 2003^7^ and another that reported an overall increase in brain tumors, during 1995–2017.^8^ Other studies of temporal trends in incidence in the United Kingdom have been more limited in scope, and restricted to particular CNS tumor subtypes, specific geographic regions, age groups, or shorter time periods.^9–15^

Diagnosis of a CNS tumor may be achieved through various methods. Microscopic confirmation (MC) of a tumor, occurs when tumor tissue is analyzed by a neuropathologist following surgical resection or biopsy. Radiographic confirmation (RC) occurs via neuroimaging such as computerized tomography (CT) or magnetic resonance imaging (MRI). This is accepted in clinical practice when the radiological findings are compelling and immediate neurosurgical intervention is not required, or when surgery/biopsy carries substantial risks and does not affect clinical management.^3^ Recent studies in the United States and Wales have shown distinct trends in incidence rates by the method of diagnosis and have suggested that future reports of incidence trends should provide information on the method of diagnosis to avoid misinterpretation of the data.^16,17^ None of the aforementioned English studies quantified temporal trends by the method of diagnosis.

The aim of this study was to characterize temporal trends in the incidence of primary CNS tumors diagnosed in England during a recent 25-year period (1993–2017) and to investigate factors that might have contributed to the observed trends.

## Methods

### Study Design and Data Sources

This population-based cohort study used data from the National Cancer Registration and Analysis Service (NCRAS)^18^ and included all individuals diagnosed with a primary CNS tumor in England between January 1st, 1993 and December 31st, 2017. NCRAS registers all malignant and selected nonmalignant neoplasms in all individuals resident in England. Tumor behavior was determined according to the 5th digit of the International Classification of Diseases for Oncology Third Edition (ICD-O-3) morphology code, which indicates whether a tumor exhibits malignant, benign, or uncertain behavior. Tumor anatomical locations were identified according to the International Classification of Diseases 9th (ICD-9) and 10th Revision (ICD-10) topography codes. Specially trained clinical coders within NCRAS code the data before entering it into the cancer registration system.

Data on CT head and MRI brain scans performed within the National Health Service (NHS), which is the publicly funded healthcare system in England, were available for the period 2013–2017 from the Diagnostic Imaging Dataset via NHS Digital. These data were categorized according to the following patient settings: Accident & emergency, inpatient, general practitioner request, and outpatient.

### Method of Diagnosis

Within NCRAS, the method of diagnosis is determined using the 9 categories outlined in ICD-O-3 (Supplementary Table 1).^19^

In this study, the term microscopically confirmed (MC) indicates that the diagnosis was based on histological examination of tissue from a primary tumor and radiographically confirmed (RC) indicates that the diagnosis was based on imaging. The remaining 7 categories were combined together as “Other.”

### Inclusion Criteria

Primary CNS tumors were identified using topography codes for ICD-10 (C70, C71, C72, C75.1–C75.3, D32, D33, D35.2–D35.4, D42, D43, and D44.3–D44.5) and ICD-9 (191, 192, 194.3–194.4, 225, 227.3–227.4, 237.0–237.1, 237.6, and 237.9) (Supplementary Table 2). Secondary (metastatic) CNS tumors were excluded, as were individuals missing key cancer registration variables (age, sex, date of diagnosis, tumor behavior, and tumor location; <0.0001% of all cases).

### Statistical Analyses

Age-sex-standardized annual incidence rates per 100 000 person-years (ASR) were calculated by calendar year both overall and stratified by tumor behavior, tumor location, quintiles of the index of multiple deprivation (IMD)^20^ (a measure of socioeconomic status in England), and method of diagnosis. Annual rates of CT head and MRI brain scans were also calculated per 100 000 person-years. Denominators used annual mid-year population estimates obtained from the Office for National Statistics. Standardization was to the 2013 European Standard Population. Trends for males and females were similar, so we present results for the sexes combined.

Age-specific annual incidence rates, standardized for sex, were calculated for the entire study period using 5-year age groups, stratified by the method of diagnosis, tumor behavior, and tumor location. To examine age-specific temporal trends, due to smaller numbers, 5 broader age groups (pediatric—0 to 14, teenagers and young adults (TYA)—15 to 24, adults—25 to 64, older people—65 to 84, and elderly—85+ years) were used. Incidence rates were age-sex-standardized by 5-year intervals within these broad age groups.

Temporal trends were quantified by estimating the average annual percent change (AAPC) in the incidence rates and their 95% confidence intervals using joinpoint regression models.^21^ To compute this, the best fitting regression model using joined log-linear segments is selected to identify calendar years during which there was a significant change in the annual percent change for each segment. A single AAPC to describe the average change over the entire 25-year study period is then estimated by taking a weighted average of the slope coefficients of each individual annual percent change segment from the regression model. The AAPC estimate is a useful summary measure that allows for the comparison of trends.

Analyses were performed using Stata 17.0 or Joinpoint 4.9.1.0.

### Ethical Approval

This study was approved by the London–London Bridge Research Ethics Committee, NHS Health Research Authority. Informed consent was not required as this study used de-personalized data, collected via routine methods by NCRAS. This study was conducted in accordance with the principles of the Declaration of Helsinki.

## Results

### Characteristics of the Study Population

During the 25-year study period (1993–2017), 188 340 individuals were diagnosed with a primary CNS tumor in England (Table 1). The annual case count rose from 5571 in 1993 to 9835 in 2017. Malignant tumors accounted for 52% of all CNS tumors, while 37% were benign and 11% were of uncertain behavior (Supplementary Table 3). The majority of CNS tumors were located in the brain (58%) or meninges (22%), while the remainder were in the endocrine glands (11%) or in the spinal cord and other parts of the CNS (9%). Over the study period, the percentage of diagnoses that were microscopically confirmed (MC) each year, varied only between 62% and 70% with no trend. In contrast, the percentage that were radiographically confirmed (RC) each year rose steadily and by a much larger amount, from 10% in 1993 to 35% in 2017. The median age at diagnosis was 61 years (IQR: 46–73). Individuals with RC diagnoses or diagnosed by other methods tended to be older (median ages: 74, 73 IQRs: 61–82, 60–81 years) than those with MC diagnoses (median age: 55; IQR: 41–66 years). Males accounted for 50% of all diagnoses, 52% of MC diagnoses, and 44% of RC diagnoses.

### Temporal Trends Overall, and Separately by Tumor Behavior, Method of Diagnosis, Age, and Index of Multiple Deprivation

**Figure 1:**
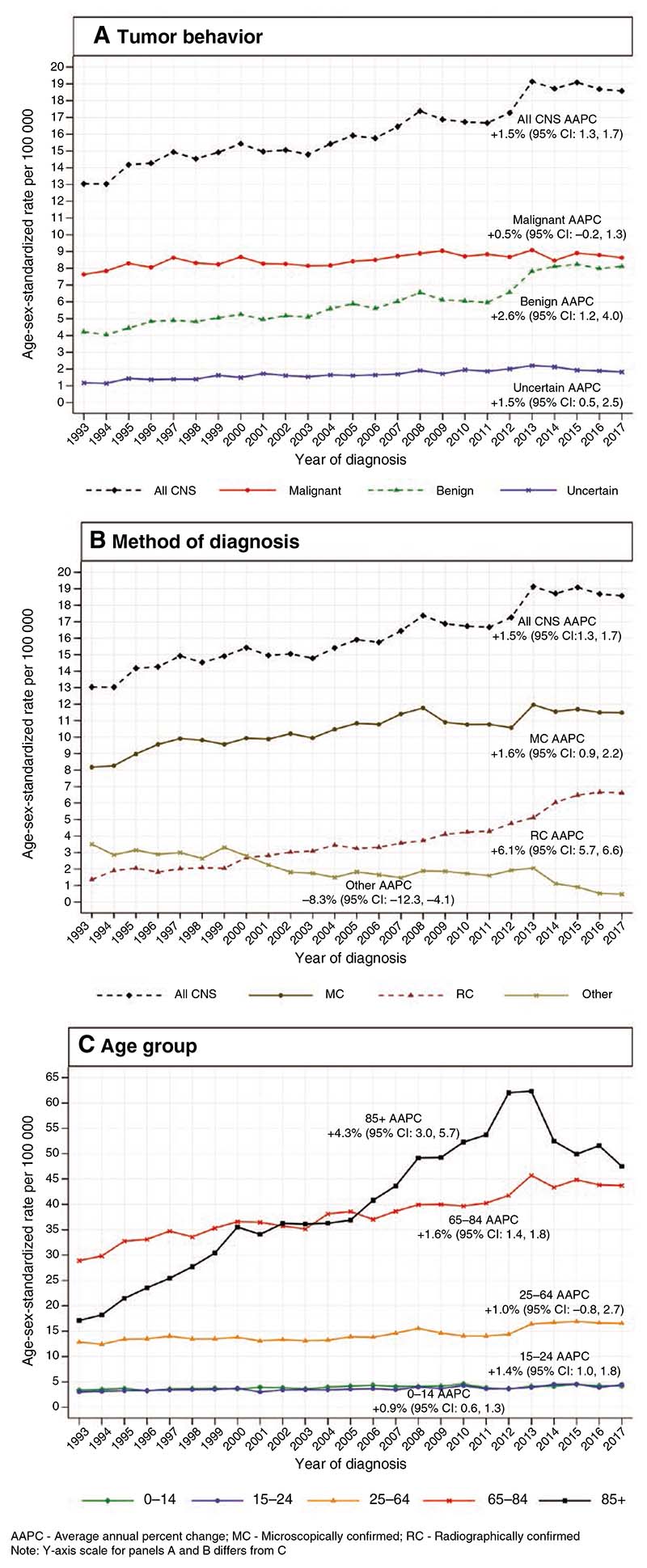
Incidence of primary central nervous system (CNS) tumors by (**A**) tumor behavior, (**B**) method of diagnosis, and (**C**) age group—England, 1993–2017.

The ASR for all CNS tumors increased from 13.0 in 1993 to 18.6 in 2017 (Figure 1; Table 2). This was chiefly due to an increase in the ASR for benign tumors, which rose steadily from 4.2 in 1993 to 8.1 in 2017 (AAPC: +2.6%, 95% CI: 1.2, 4.0). In contrast, the ASR for malignant tumors changed only from 7.6 in 1993 to 8.6 in 2017 (AAPC: +0.5%, 95% CI: −0.2, 1.3), while the ASR for tumors of uncertain behavior increased modestly from 1.2 in 1993 to 1.8 in 2017 (AAPC: +1.5%, 95% CI: 0.5, 2.5).

Considering the method of diagnosis, the ASR for all MC tumors increased from 7.4 in 1993 to 11.0 in 2017 (AAPC: +1.6%, 95% CI: 0.9, 2.2). The ASR for all RC tumors rose by a similar absolute amount, from 1.2 in 1993 to 6.2 in 2017, but constituted a larger relative change (AAPC: +6.1%, 95% CI: 5.7, 6.6). Meanwhile, the ASR for tumors diagnosed via other methods decreased from 3.0 in 1993 to 0.4 in 2017 (AAPC: −8.3%, 95% CI −12.3, −4.1).

For pediatric tumors and tumors in TYA, the ASRs rose by small absolute amounts (pediatric: 3.4–4.2; TYA: 3.0–4.5) which were, nevertheless, significant (pediatric AAPC: +0.9%, 95% CI: 0.6, 1.3; TYA AAPC: +1.4%, 95% CI: 1.0, 1.8). Meanwhile, for adults aged 25–64 years, the ASR rose from 12.9 in 1993 to 16.6 in 2017, which was not significant (AAPC: +1.0, 95% CI: −0.8, 2.7). In contrast, the ASR for tumors in older people aged 65–84 years increased by a large absolute amount from 28.9 in 1993 to 43.7 in 2017 (AAPC: +1.6%, 95% CI: 1.4, 1.8). In those who are aged 85+ years, the ASR increased by an even larger absolute amount, from 17.1 in 1993 to 47.5 in 2017 (AAPC: +4.3%, 95% CI: 3.0, 5.7).

To see whether the large increases in the ASRs of RC tumors in older age groups were associated with socioeconomic status, we computed ASRs by age at diagnosis, method of diagnosis, and index of multiple deprivation (Supplementary Figure 2). There were some differences in the ASRs according to the index of multiple deprivation quintiles, but no evidence that these differences change substantially with the calendar year.

### Temporal Trends by Tumor Behavior

**Figure 2:**
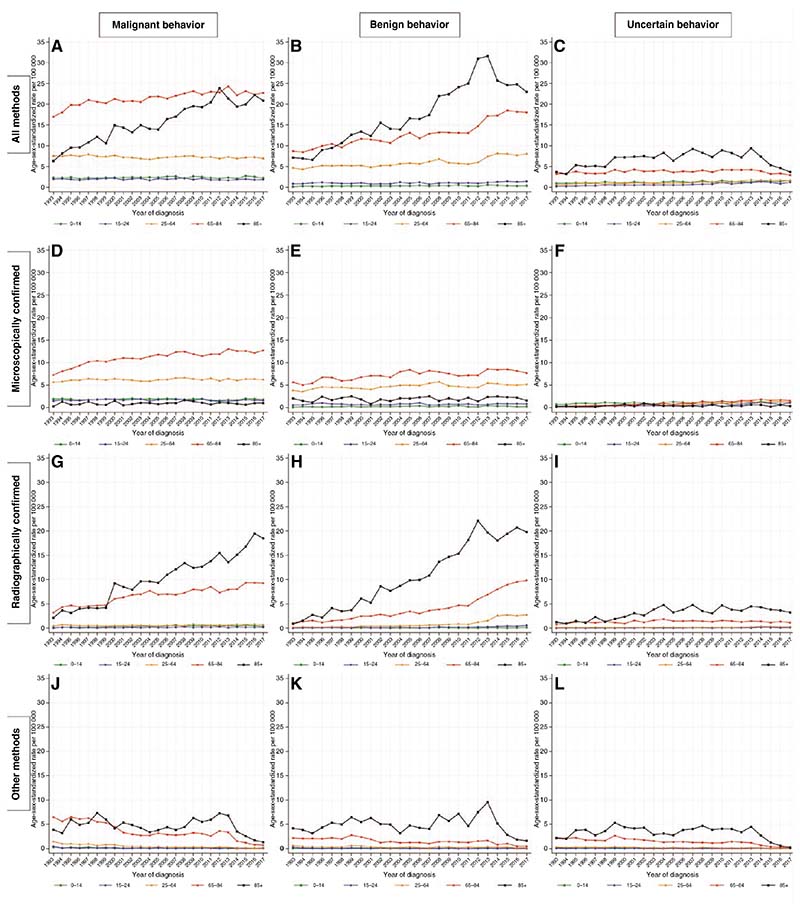
Incidence of primary central nervous system (CNS) tumors by tumor behavior, method of diagnosis and age group—England, 1993–2017.

The large absolute increase in the ASR for those who are aged 85+ years was primarily accounted for by large increases in the ASR for all malignant RC tumors, where the ASR increased from 2.1 in 1993 to 18.5 in 2017 (AAPC: +8.9%, 95% CI: 6.8, 11.0), and for all benign RC tumors where the ASR increased from 0.9 in 1993 to 19.8 in 2017 (AAPC: +9.4%, 95% CI: 7.9, 10.9) (Figure 2). The ASR for all tumors of uncertain behavior in this age group (85+ years) did not change significantly, while the ASR for tumors diagnosed by other methods decreased irrespective of tumor behavior.

Considerable absolute increases were also seen in the ASRs for those aged 65–84 years for all malignant MC tumors, where the ASR increased from 7.3 in 1993 to 12.7 in 2017 (AAPC: +2.4%, 95% CI 1.8, 3.0), and for all malignant RC tumors where the ASR increased from 3.2 in 1993 to 9.3 in 2017 (AAPC: +3.9%, 95% CI 2.9, 4.9). Similar trends were seen for benign MC tumors which increased from 5.7 in 1993 to 7.7 in 2017 (AAPC: +1.6, 95% CI: 0.8,2.5) and benign RC tumors which increased from 0.9 in 1993 to 9.8 in 2017 (AAPC: +9.5%, 95% CI 8.3, 10.8). The ASRs for MC and RC tumors of uncertain behavior were small and did not contribute materially to overall trends. For tumors diagnosed by other methods, the ASRs decreased during the study period in this age group irrespective of tumor behavior.

For adults aged 25–64 years, for teenagers and young adults aged 15–24, and for pediatric tumors in ages 0–14, there were significant increases for some categories, most notably for benign RC tumors in 25–64-year-olds which increased from 0.2 in 1993 to 2.7 in 2017 (AAPC: +11.0%, 95% CI 8.5, 13.6). However, this and other increases were balanced by several decreases, especially for tumors diagnosed by other methods.

### Temporal Trends by Anatomical Location

**Figure 3:**
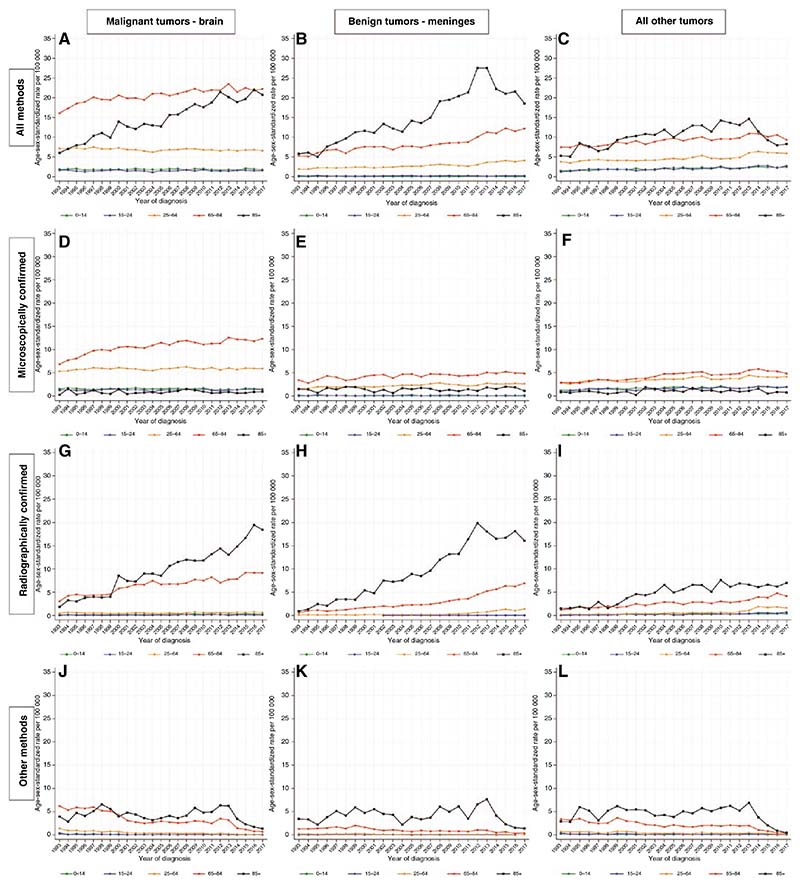
Incidence of primary central nervous system (CNS) tumors by anatomical location, method of diagnosis and age group—England, 1993–2017.

The commonest anatomical location for malignant tumors was the brain (*n* = 92 250; 49% of all CNS tumors), and the commonest anatomical location for benign tumors was the meninges (*n* = 37 806; 20% of all CNS tumors). For both of these categories, there were striking absolute increases in the ASR for individuals aged 85+ years. This was due to the predominant increases in the ASR for RC tumors, from 1.9 in 1993 to 18.4 in 2017 (AAPC: +9.5%, 95% CI: 7.6, 11.5) for the former, and from 0.8 in 1993 to 16.1 in 2017 (AAPC: +9.3% (95% CI: 7.7, 10.8) for the latter (Figure 3; Supplementary Table 5A). For malignant brain tumors, there have also been considerable absolute increases at ages 65–84 years in both MC and RC tumors, no significant changes at ages 0–14, 15–24, and 25–64 years in MC tumors, and significant increases at ages 0–14 and 25–64 years in RC tumors. Over the same period, there have been significant decreases in malignant brain tumors diagnosed by other methods in all age groups. For benign tumors of the meninges, there have been substantial increases in RC tumors at ages 65–84 from 0.4 in 1993 to 6.9 in 2017 (AAPC: +9.0%, 95% CI: 6.5, 11.5), and at ages 25–64 from 0.1 in 1993 to 1.4 in 2017 (AAPC: +9.9%, 7.7, 12.1). There were also significant but numerically smaller increases in benign tumors of the meninges at ages 65–84 and 25–64 years, and a significant decrease in tumors diagnosed by other methods at ages 65–84 years.

For tumors diagnosed in all other combinations of behavior and anatomical location combined, there were significant increases in MC tumors in all age groups under 65 years, with AAPCs ranging from +1.5 to +2.1%, while RC tumors increased significantly in all age groups, with AAPCs ranging from +4.5 to +8.6%. During the same period, tumors diagnosed via other methods significantly decreased in all age groups, with AAPCs ranging from −3.2 to −9.2% (see Supplementary Table 5a–d and Supplementary Figures 3–5 for data on all combinations of behavior and anatomical location separately).

### Imaging Trends (CT Head and MRI Brain Scans) in England, 2013–2017

**Figure 4:**
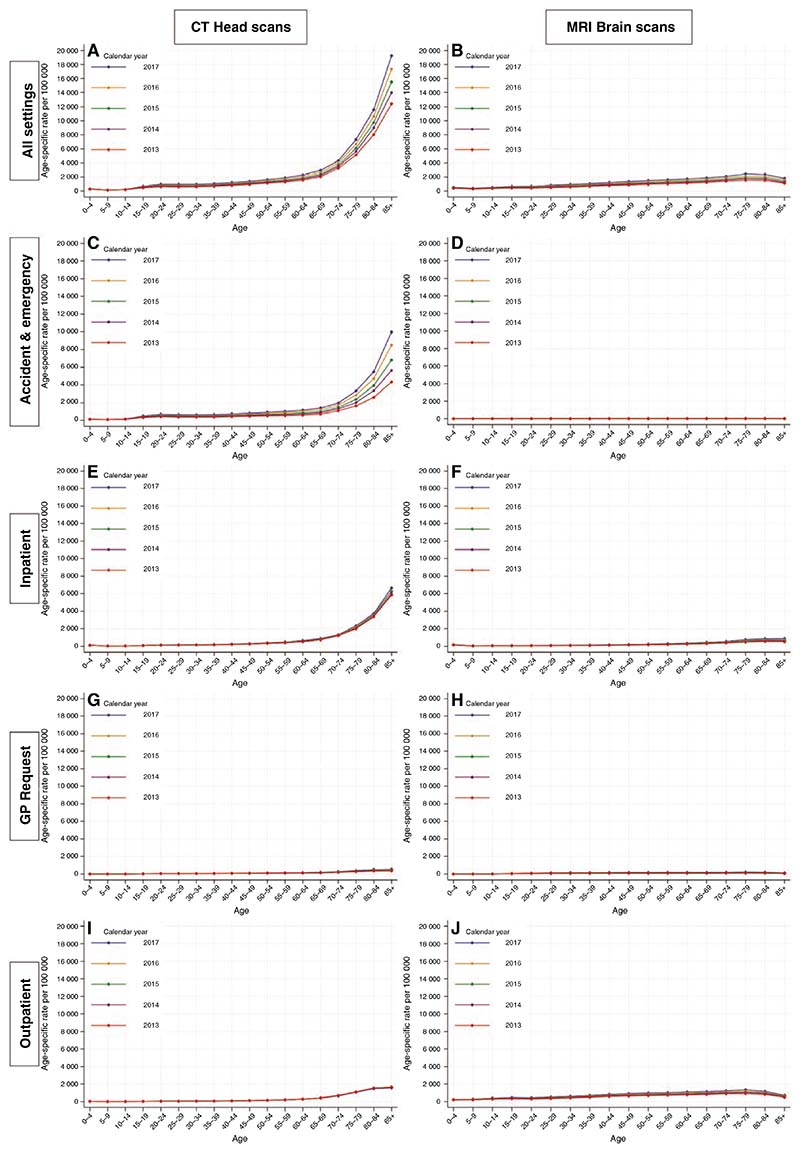
Age-specific rates of computerized tomography (CT) head and magnetic resonance imaging (MRI) brain scans by patient setting—England, 2013–2017.

During the 5 years spanning 2013–2017, there were a total of 5 012 335 CT head scans and 2 676 420 MRI brain scans performed within the NHS in England. For CT head scans performed in England across all patient settings combined, rates were higher at older ages (Figure 4). There were also striking increases in the rate of scans in those aged 65 years and above, over each calendar year between 2013 and 2017. The most notable increase was observed in individuals aged 85+ years, for whom the rate rose from 12 409 scans per 100 000 in 2013 to 19 232 scans per 100 000 in 2017.

Most of the increase in the calendar year was due to CT head scans performed in Accident & Emergency (A&E) settings, especially in those aged 65–84 and 85+ years, where the rate of scans per 100 000 approximately doubled between 2013 and 2017, from 1312 to 2613 and from 4349 to 9984 respectively. Inpatient CT head scans also increased substantially with advancing age, but these rates have remained largely stable over each calendar year. Meanwhile, there were very low rates of scans performed due to a general practitioner request or those in an outpatient setting, with slight increases after the age of 65 years, but little change over the calendar year.

Considering MRI brain scans, the absolute rates were substantially lower than those for CT head scans and there was little change according to age or calendar year.

## Discussion

### Summary of Findings

We observed a modest increase in the overall incidence rate of primary CNS tumors in England between 1993 and 2017, using national cancer registry data. Increases were substantially greater for benign tumors compared to tumors of malignant or uncertain behavior, for RC diagnoses compared to MC diagnoses, and for older individuals aged 65–84 and 85+ years compared to younger people. The most notable increase was in the incidence rate of RC benign tumors of the meninges. Meanwhile, incidence rates for malignant CNS tumors have remained stable over the 25-year study period.

For most cancers, MC is regarded as the most accurate method of diagnosis and, together with very low proportions of “death certificate only (DCO)” or “unknown” diagnoses, indicates high data quality within a cancer registry. In the context of CNS tumors, however, MC involves complex neurosurgical intervention such as brain surgery or biopsy. This carries substantially greater risks than similar procedures for other organs, and will only be conducted if the benefits are likely to outweigh the risks. Determining tumor histology via MC can, however, be an important prerequisite for administering appropriate treatment, as histology can predict response to radiotherapy and chemotherapy.

In older age groups, RC was more common than MC, perhaps reflecting shifts in the risk-benefit ratio with increasing age. Surgery may be performed less often in older patients due to poorer health, comorbidities, reduced functional status, and the inherent risks associated with major cranial surgery.^22^ Furthermore, older patients are more likely to undergo brain scans for falls or other indications, and so a greater proportion of diagnoses could arise incidentally. Support for this theory is provided by our analysis of national imaging data, which revealed considerable increases in the rate of CT head scans performed in England over a recent 5-year period (2013–2017), particularly among individuals aged at least 65 years attending A&E departments. Although such scans are often used to diagnose brain and other CNS tumors, they do have wider clinical uses and we are unable to determine the rationale behind each individual scan. In clinical practice across hospitals in England, there is a low threshold for requesting CT head scans in older patients presenting with common neurological symptoms or deficits. CT scans are a convenient imaging modality that can be used quickly to rule out serious structural brain lesions such as bleeds, strokes, and tumors. The benefit of using CT scans in this emergency setting outweighs any concerns about radiation exposure to patients, hence its widespread use. The substantial volume of CT head scans may result in increased incidental findings of tumors that are small or in inoperable locations.

### Findings in Context

Globally, the majority of incidence studies include only malignant CNS tumors since the registration of nonmalignant CNS tumors is limited or nonexistent in many regions. Over time, the registration of nonmalignant CNS tumors has improved due to increasing recognition of the serious impact of these tumors on both individuals and healthcare systems. A recent study of 96 population-based registries in 39 countries reported a five-fold difference in the incidence of malignant CNS tumors between the highest incidence registries, mainly in Europe, and the lowest incidence registries, mainly in Asia.^23^

There is also notable global variation in temporal trends in CNS tumor incidence. Population-based studies from regions in Europe, the United States, and China have reported increasing incidence rates of all CNS tumors,^6,8,24–28^ consistent with the findings of our study, while others, in Italy and the Nordic countries have reported stable,^7,29–32^ or in the case of Japan, decreasing incidence rates.^33^ When restricted to malignant CNS tumors, the majority of studies report stable^26,28,34–38^ or decreasing incidence rates,^2,39–41^ while some studies report increasing incidence rates over time.^42,43^ Among the few studies of nonmalignant CNS tumors, increasing rates have been reported in parts of the United States, Wales, and Spain,^16,17,28^ which is consistent with our study, but stable rates have been reported in another US study, across Nordic countries, and Australia.^2,35,38^

### CNS Tumor Incidence Trends in England

Two large studies from England have reported inconsistent trends in the incidence of CNS tumors. A study covering the period 1979–2003 reported an increase in the incidence of CNS tumors during 1979–1992, followed by a leveling off during 1993–2003.^7^ Authors found the early increases were mainly in the young (0–14 years, AAPC: +1.3%) and elderly (65–84 years, AAPC: +2.5%). A more recent study of brain tumors in adults in England during 1995–2017 reported an overall increase, but did not quantify the observed trends.^8^ Both studies reported variation in the temporal trends by subtype, and hypothesized that increases may be attributed to the emergence and availability of neuroimaging, advances in clinical practice, diagnostic specificity, and improvements in cancer registration practices. Our quantification of temporal trends by the method of diagnosis illustrates greater increases in the incidence rates of RC tumors over time, particularly in older people, and provides evidence consistent with changes in neuroimaging and clinical practice.

### Temporal Trends by the Method of Diagnosis and Tumor Behavior

When not accounting for the method of diagnosis, the increase in the incidence rate for all CNS tumors combined found in our study (AAPC: +1.5%, 1993–2017) is largely consistent with studies in Wales^17^ (AAPC: +1.6%, 1997–2015) and Australia^44^ (AAPC: +1.2%, 2000–2008), but lower than studies in the US SEER^17^ (AAPC: +1.9%, 2004–2015), regions within Spain^28^ (AAPC: +2.1% 1994–2013) and France^25^ (AAPC: +2.7% 2000–2012), and higher than those who reported from Nordic countries^31^ (AAPC: +0.6% to +0.9%, 1969–1998). In Japan,^33^ CNS tumor incidence rates were increasing during 1975–1987 (AAPC: +3.1%), but have been reported to be declining during 1987–2004 (AAPC: −1.8%).

Few studies have investigated incidence rate trends according to both methods of diagnosis and tumor behavior. For CNS tumors of all behaviors, MC incidence rates increased more rapidly in Wales^17^ (AAPC: +3.6%, 1997–2015) than in the United States^17^ (AAPC: +0.1%, 2004–2015) or our study in England (AAPC: +1.6%, 1993–2017). Data on RC diagnoses are very limited. We showed substantial increases in rates of RC tumors of all behaviors (AAPC: +6.1%, 1993–2017) which were greater than a recent study during a similar period from Girona, Spain^28^ (AAPC: +3.9%, 1994–2013). When restricted to malignant tumors, for MC diagnoses, our study (AAPC: +1.0%, 1993–2017) found an opposite trend to a study over a similar period from Finland^45^ (AAPC: −0.9%, 1990–2016).

In the United States,^16^ rates of nonmalignant MC tumors have decreased (AAPC: −1.9% to −0.3%, 2004–2017) which differs from the increasing trends observed in our study (AAPC: +1.3%, 1993–2017). Meanwhile, we showed higher increases in rates of RC benign tumors (AAPC: +10.2%, 1993–2017) compared to the US^16^ (AAPC: +1.7% to +2.3%, 2004–2017). While the central brain tumor registry of the United States (CBTRUS)^2^ and a US study^16^ using the SEER database reported high increases in rates of RC nonmalignant tumors during an earlier period (AAPC: +9.9% and +9.5%, 2004–2009), these increases have attenuated more recently (AAPC: +1.8% and +2.3%, 2009–2018 and 2010–2017). An increase in rates of RC diagnoses and nonmalignant CNS tumors in the data may reflect better data collection over time. In 1998, the European Network of Cancer Registries Working Group on Brain and nervous system tumors recommended that all cancer registries include all intracranial and intraspinal neoplasms irrespective of their behavior.

### Strengths and Limitations

NCRAS has collected high-quality data on all cancers and on nonmalignant CNS tumors diagnosed each year in England throughout the study period. Data are collected from multiple sources ensuring all avenues are exhausted to capture a complete dataset of diagnosed cases. Full details on the structure and robustness of the NCRAS dataset have been published previously.^18^ Our study, which covers 25 years, is the longest-spanning study to systematically investigate the influence of the method of diagnosis on temporal trends in CNS tumor incidence rates. While other studies have alluded to the increased availability of neuroimaging as a potential explanation for increasing incidence rates, few have examined trends by the method of diagnosis. By doing so, this study may contribute to our understanding of the role of the method of diagnosis on temporal trends in CNS tumor incidence.

As our study is based on routinely collected data, we are reliant on data being input and coded correctly. With respect to the completeness, only NHS healthcare providers are currently mandated to submit data to NCRAS. While some diagnoses occurring under private healthcare may be missed, it is estimated that the NHS funds 98–99% of hospital activity^46^ and thus we may assume near complete coverage of CNS tumor diagnoses in England.

Although a lower proportion of MC diagnoses may be seen as a limitation since they are regarded as more reliable due to being based on tumor histology, an increase in RC diagnoses could indicate that case ascertainment has improved over time, thus capturing a wider range of tumors. Interpretation of temporal trends should therefore be considered in context, as an increase in RC diagnoses will include some tumors that were found incidentally upon scanning for another indication, and without which they might never have been identified during the individual’s lifetime. Currently, little is known regarding the implications of these incidental diagnoses in terms of subsequent patient morbidity and mortality as it is not possible to distinguish between incidental and symptomatic diagnoses in the data available to us.

## Conclusion

Overall increases in the incidence rates of CNS tumors in England may be attributed to greater detection of benign tumors, particularly those in the meninges and those that are radiographically confirmed. This could be due to the more widespread use of neuroimaging, particularly CT head scans performed in older people aged at least 65 years in A&E departments, in addition to improved registration practices. The incidence of malignant brain tumors, which comprise over 50% of all CNS tumors, has remained relatively stable over the past 25 years.

Due to rates of RC diagnoses increasing rapidly over time, we recommend all future studies of incidence trends report results according to the method of diagnosis, where the source of data allows. This will help provide a better understanding of temporal trends and allow clinically meaningful interpretations and comparisons to be made, leading to a clearer picture of the true burden of this disease.

## Supplementary Material

Supplementary Material

## Figures and Tables

**Table 1:** Patient and Tumor Characteristics of 188 340 Individuals Diagnosed With a Primary CNS Tumor According to the Method of Diagnosis—England, 1993–2017 (Row Percentages Presented—See Supplementary Table 3 for Column Percentages)

Characteristic	MC		RC		Other		Total	
	*n*	%	*n*	%	*n*	%	*n*	%
Sex								
Male	64 124	69	18 679	20	10 444	11	93 247	100
Female	60 066	63	23 378	25	11 649	12	95 093	100
Age group at diagnosis								
Pediatric (0–14)	7600	83	1197	13	404	4	9201	100
Teenagers and young adults (15–24)	4890	83	642	11	393	7	5925	100
Adults (25–64)	75 596	82	10 530	11	6066	7	92 192	100
Older people (65–84)	35 252	50	22 843	33	11 847	17	69 942	100
Elderly (85+)	852	8	6845	62	3383	31	11 080	100
Age at diagnosis								
Median (IQR)	55	(41–66)	74	(61–82)	73	(60–81)	61	(46–73)
Calendar year of diagnosis								
1993	3569	64	558	10	1444	26^a^	5571	100
1994	3630	65	772	14	1167	21	5569	100
1995	3949	65	835	14	1305	21	6089	100
1996	4208	69	746	12	1186	19	6140	100
1997	4369	68	836	13	1244	19	6449	100
1998	4381	69	864	14	1092	17	6337	100
1999	4283	66	849	13	1383	21	6515	100
2000	4475	66	1132	17	1181	17	6788	100
2001	4478	68	1195	18	952	14	6625	100
2002	4646	69	1289	19	769	11	6704	100
2003	4569	69	1328	20	752	11	6649	100
2004	4841	69	1494	21	647	9	6982	100
2005	5061	69	1422	20	808	11	7291	100
2006	5091	70	1474	20	739	10	7304	100
2007	5407	70	1608	21	659	9	7674	100
2008	5645	69	1701	21	864	11	8210	100
2009	5284	66	1908	24	865	11	8057	100
2010	5304	66	1988	25	805	10	8097	100
2011	5367	66	2023	25	756	9	8146	100
2012	5312	62	2307	27	921	11	8540	100
2013	6081	63	2511	26	1009	11	9601	100
2014	5951	62	3031	32	564	6	9546	100
2015	6102	62	3289	33	462	5	9853	100
2016	6063	62	3433	35	272	3	9768	100
2017	6124	62	3464	35	247	3^b^	9835	100
Tumor behavior^c^								
Malignant	66 684	68	20 280	21	11 067	11	98 031	100
Benign	46 033	66	17 076	25	6249	9	69 358	100
Uncertain	11 473	55	4701	22	4777	23	20 951	100
Anatomical location^d^								
Meninges (C70.0–C70.9, D32.0–D32.9, D42.0–D42.9)	26 604	63	11 518	27	4121	10	42 243	100
Brain (C71.0–C71.9, D33.0–D33.2, D43.0–D43.2)	70 620	65	23 447	22	14 325	13	108 392	100
Spinal cord and other CNS (C72.0–C72.9, D33.3–D33.9, D43.3–D43.9)	12 318	71	3466	20	1545	9	17 329	100
Endocrine CNS (C75.1–C75.3, D35.2–D35.4, D44.3–D44.5)	14 648	72	3626	18	2102	10	20 376	100
Tumor behavior according to anatomical location^cd^								
Malignant								
Meninges	1129	65	332	19	276	16	1737	100
Brain	62 760	68	19 224	21	10 266	11	92 250	100
Spinal cord and other CNS	1948	70	552	20	295	11	2795	100
Endocrine Glands in CNS	847	68	172	14	230	18	1249	100
Benign								
Meninges	23 194	61	10 936	29	3676	10	37 806	100
Brain	1842	67	559	20	335	12	2736	100
Spinal cord and other CNS	8996	71	2652	21	1012	8	12 660	100
Endocrine glands in CNS	12 001	74	2929	18	1226	8	16 156	100
Uncertain								
Meninges	2281	84	250	9	169	6	2700	100
Brain	6018	45	3664	27	3724	28	13 406	100
Spinal cord and other CNS	1374	73	262	14	238	13	1874	100
Endocrine glands in CNS	1800	61	525	18	646	22	2971	100
Total (all CNS tumors)	124 190	66	42 057	22	22 093	12	188 340	100

**Abbreviation:** CNS, central nervous system; MC, microscopically confirmed; RC, radiographically confirmed.

a In 1993, “Other” comprised: Death Certificate Only-4%; Clinical-14%; Other special tests/Specific tumor marker/Cytology/Histology of metastases-<1%; Unknown-7%

b In 2017 “Other” comprised: Death Certificate Only-0%; Clinical-1%; Other special tests/Specific tumor marker/Cytology/Histology of metastases-<1%; Unknown-1%

c Behavior based on the 5th digit of the ICD-O-3 histology code.

d Anatomical location based on the ICD-10 topography code.

**Table 2:** Age-Sex-Standardized^a^ Incidence Rates (ASR) Per 100 000 for Calendar Years 1993 and 2017 Separately and Average Annual Percentage Change (AAPC) for 188 340 Individuals Diagnosed With a Primary CNS Tumor According to Tumor Behavior^b^ and Method of Diagnosis—England, 1993–2017

Characteristic	Method of diagnosis	1993 only				2017 only				1993–2017				*P*-Value
		*n*	%	ASR	95% Cl	*n*	%	ASR	95% CI	*n*	%	AAPC	95% CI	
All CNS tumors														
All ages	All	5571	100	13.0	(12.7, 13.4)	9835	100	18.6	(18.2, 18.9)	188 340	100	1.5*	(1.3, 1.7)	<.001
	MC	3569	64	7.4	(7.9, 8.5)	6124	62	11.0	(11.2, 11.8)	124 190	66	1.6*	(0.9, 2.2)	<.001
	RC	558	10	1.2	(1.2, 1.5)	3464	35	6.2	(6.4, 6.8)	42 057	22	6.1*	(5.7, 6.6)	<.001
	Other	1444	26	3.0	(3.3, 3.7)	247	3	0.4	(0.4, 0.5)	22 093	12	−8.3*	(−12.3, −4.1)	<.001
All CNS tumors														
Pediatric (0–14)	All	315	100	3.4	(3.0, 3.7)	422	100	4.2	(3.8, 4.6)	9201	100	0.9*	(0.6, 1.3)	<.001
	MC	252	80	2.7	(2.4, 3.0)	341	81	3.4	(3.0, 3.7)	7600	83	0.7*	(0.3, 1.1)	.002
	RC	18	6	0.2	(0.1, 0.3)	69	16	0.7	(0.5, 0.8)	1197	13	5.8*	(3.4, 8.3)	<.001
	Other	45	14	0.5	(0.3, 0.6)	12	3	0.1	(0.1, 0.2)	404	4	−5.3*	(−6.8, −3.9)	<.001
Teenagers and young adults (15–24)	All	193	100	3.0	(2.6, 3.5)	298	100	4.5	(4.0, 5.0)	5925	100	1.4*	(1.0, 1.8)	<.001
	MC	140	73	2.2	(1.8, 2.6)	235	79	3.5	(3.1, 4.0)	4890	83	1.2*	(0.8, 1.7)	<.001
	RC	4	2	0.1	(0.0, 0.2)	59	20	0.9	(0.7, 1.1)	642	11	6.4*	(4.8, 8.0)	<.001
	Other	49	25	0.8	(0.6, 1.0)	4	1	0.1	(0.0, 0.2)	393	7	−4.6*	(−6.8, −2.3)	<.001
Adults (25–64)	All	2936	100	12.9	(12.4, 13.3)	4688	100	16.6	(16.1, 17.0)	92 192	100	1.0	(−0.8, 2.7)	.283
	MC	2247	77	9.8	(9.4, 10.2)	3598	77	12.7	(12.3, 13.1)	75 596	82	0.9*	(0.6, 1.2)	<.001
	RC	167	6	0.7	(0.6, 0.8)	1025	22	3.6	(3.4, 3.9)	10 530	11	6.6*	(3.3, 10.0)	<.001
	Other	522	18	2.3	(2.1, 2.5)	65	1	0.2	(0.2, 0.3)	6066	7	−6.5*	(−7.8, −5.3)	<.001
Older people (65–84)	All	1983	100	28.9	(27.6, 30.2)	3783	100	43.7	(42.3, 45.1)	69 942	100	1.6*	(1.4, 1.8)	<.001
	MC	909	46	13.2	(12.3, 14.1)	1911	51	22.0	(21.0, 23.0)	35 252	50	2.2*	(0.6, 3.9)	.008
	RC	331	17	4.8	(4.3, 5.4)	1748	46	20.2	(19.3, 21.2)	22 843	33	5.6*	(3.4, 7.8)	<.001
	Other	743	37	10.9	(10.1, 11.7)	124	3	1.4	(1.2, 1.7)	11 847	17	−8.7*	(−10.9, −6.5)	<.001
Elderly (85+)	All	144	100	17.1	(14.3, 20.0)	644	100	47.5	(43.8, 51.2)	11 080	100	4.3*	(3.0, 5.7)	<.001
	MC	21	15	2.6	(1.5, 3.7)	39	6	2.8	(1.9, 3.7)	852	8	0.7	(−0.3, 1.7)	.184
	RC	38	26	4.4	(3.0, 5.9)	563	87	41.5	(38.1, 45.0)	6845	62	9.8*	(8.2, 11.4)	<.001
	Other	85	59	10.2	(8.0, 12.5)	42	7	3.2	(2.2, 4.1)	3383	31	−5.2*	(−8.1,−2.3)	.001
Malignant behavior														
Pediatric (0–14)	All	216	100	2.3	(2.0, 2.6)	223	100	2.2	(1.9, 2.5)	5344	100	0.0	(−0.4, 0.5)	.885
	MC	177	82	1.9	(1.6, 2.2)	182	82	1.8	(1.5, 2.0)	4220	79	−0.4	(−0.8, 0.0)	.062
	RC	12	6	0.1	(0.1, 0.2)	36	16	0.4	(0.2, 0.5)	880	16	4.1*	(1.6, 6.7)	.001
	Other	27	13	0.3	(0.2, 0.4)	5	2	0.1	(0.0, 0.3)	244	5	−3.9*	(−5.9,−1.9)	.001
Teenagers and young adults (15–24)	All	124	100	2.0	(1.6, 2.3)	123	100	1.8	(1.5, 2.2)	2855	100	0.1	(−0.4, 0.6)	.605
	MC	94	76	1.5	(1.2, 1.8)	108	88	1.6	(1.3, 1.9)	2461	86	0.3	(−0.2, 0.8)	.264
	RC	2	2	0.0	(0.0, 0.1)	13	11	0.2	(0.1, 0.3)	221	8	1.7	(−0.3, 3.7)	.094
	Other	28	23	0.4	(0.3, 0.6)	2	2	0.0	(0.0, 0.1)	173	6	−5.3	(−11.4, 1.2)	.108
Adults (25–64)	All	1719	100	7.6	(7.2, 7.9)	1973	100	7.0	(6.7, 7.3)	46 167	100	−0.3	(−1.4, 0.8)	.581
	MC	1299	76	5.7	(5.3, 6.0)	1767	90	6.2	(5.9, 6.5)	39 551	86	0.4	(−0.8, 1.6)	.511
	RC	103	6	0.5	(0.4, 0.6)	187	9	0.7	(0.6, 0.8)	3648	8	1.5*	(0.6, 2.4)	.002
	Other	317	18	1.4	(1.3, 1.6)	19	1	0.1	(0.1, 0.1)	2968	6	−8.6*	(−9.7, −7.4)	<.001
Older People (65–84)	All	1166	100	17.0	(16.0, 18.0)	1966	100	22.7	(21.7, 23.7)	39 242	100	1.4*	(0.6, 2.2)	.001
	MC	504	43	7.3	(6.7, 7.9)	1105	56	12.7	(12.0, 13.5)	20 205	51	2.4*	(1.8, 3.0)	<.001
	RC	221	19	3.2	(2.8, 3.7)	798	41	9.3	(8.6, 9.9)	12 597	32	3.9*	(2.9, 4.9)	<.001
	Other	441	38	6.5	(5.9, 71)	63	3	0.7	(0.5, 0.9)	6440	16	−9.7*	(−13.1,−6.2)	<.001
Elderly (85+)	All	55	100	6.3	(4.6, 8.0)	283	100	20.9	(18.4, 23.3)	4423	100	3.7*	(2.6, 4.8)	<.001
	MC	2	4	0.3	(−0.1, 0.7)	14	5	1.0	(0.5, 1.6)	247	6	−0.2	(−1.8, 1.4)	.791
	RC	19	35	2.1	(1.2, 3.1)	251	89	18.5	(16.2, 20.8)	2934	66	8.9*	(6.8, 11.0)	<.001
	Other	34	62	3.9	(2.6, 5.3)	18	6	1.3	(0.7, 2.0)	1242	28	−5.1*	(−9.0, −0.9)	.016
Benign behavior														
Pediatric (0–14)	All	11	100	0.1	(0.1, 0.2)	37	100	0.4	(0.3, 0.5)	763	100	2.8*	(1.5, 4.1)	<.001
	MC	8	73	0.1	(0.0, 0.1)	25	68	0.3	(0.2, 0.4)	627	82	1.8*	(0.3, 3.3)	.021
	RC	0	0			10	27	0.1	(0.1, 0.2)	90	12	4.1*	(0.1, 8.2)	.046
	Other	3	27	0.0	(0.0, 0.1)	2	5	0.0	(0.0, 0.1)	46	6	−0.7	(−2.2, 0.9)	.359
Teenagers and young adults (15–24)	All	54	100	0.8	(0.6, 1.1)	95	100	1.4	(1.1, 1.7)	1673	100	1.5*	(0.6, 2.4)	.002
	MC	37	69	0.6	(0.4, 0.8)	56	59	0.8	(0.6, 1.1)	1246	74	0.3	(−0.6, 1.3)	.478
	RC	1	2	0.0	(0.0, 0.1)	38	40	0.6	(0.4, 0.8)	305	18	6.9*	(4.8, 9.1)	<.001
	Other	16	30	0.2	(0.1, 0.4)	1	1	0.0	(0.0, 0.1)	122	7	−2.6*	(−4.7, −0.5)	.018
Adults (25–64)	All	1057	100	4.6	(4.3, 4.9)	2263	100	8.0	(7.7, 8.3)	38 265	100	2.3*	(0.1, 4.5)	.038
	MC	875	83	3.8	(3.6, 4.1)	1462	65	5.2	(4.9, 5.4)	30 442	80	1.1*	(0.6, 1.5)	<.001
	RC	40	4	0.2	(0.1, 0.3)	767	34	2.7	(2.5, 2.9)	5837	15	11.0*	(8.5, 13.6)	<.001
	Other	142	13	0.6	(0.5, 0.7)	34	2	0.1	(0.1, 0.2)	1986	5	−3.2*	(−5.0,−1.3)	.002
Older people (65–84)	All	597	100	8.7	(8.0, 9.4)	1559	100	18.0	(17.1, 18.9)	23 773	100	2.9*	(1.5, 4.3)	<.001
	MC	389	65	5.7	(5.1, 6.2)	668	43	7.7	(71, 8.3)	13 188	55	1.6*	(0.8, 2.5)	<.001
	RC	59	10	0.9	(0.6, 1.1)	850	55	9.8	(9.2, 10.5)	7779	33	9.5*	(8.3, 10.8)	<.001
	Other	149	25	2.2	(1.8, 2.5)	41	3	0.5	(0.3, 0.6)	2806	12	−6.0*	(−11.3, −0.4)	.037
Elderly (85+)	All	58	100	7.1	(5.3, 9.0)	312	100	23.0	(20.4, 25.5)	4884	100	4.9*	(3.8, 6.0)	<.001
	MC	17	29	2.0	(1.1, 3.0)	22	7	1.6	(0.9, 2.3)	530	11	0.6	(−0.6, 1.9)	.319
	RC	8	14	0.9	(0.3, 1.6)	269	86	19.8	(17.4, 22.2)	3065	63	9.4*	(7.9, 10.9)	<.001
	Other	33	57	4.2	(2.7, 5.6)	21	7	1.6	(0.9, 2.3)	1289	26	−4.3	(−11.3, 3.2)	.252
Uncertain behavior														
Pediatric (0–14)	All	88	100	0.9	(0.7, 1.1)	162	100	1.6	(1.4, 1.9)	3094	100	2.0*	(1.4, 2.6)	<.001
	MC	67	76	0.7	(0.6, 0.9)	134	83	1.3	(1.1, 1.6)	2753	89	2.0*	(1.3, 2.7)	<.001
	RC	6	7	0.1	(0.0, 0.2)	23	14	0.2	(0.1, 0.3)	227	7	3.4*	(2.1, 4.7)	<.001
	Other	15	17	0.2	(0.1, 0.2)	5	3	0.0	(0.0, 0.1)	114	4	−3.3*	(−5.7, −0.9)	.008
Teenagers and young adults (15–24)	All	15	100	0.2	(0.1, 0.3)	80	100	1.2	(0.9, 1.5)	1397	100	3.7*	(2.6, 4.7)	<.001
	MC	9	60	0.1	(0.0, 0.2)	71	89	1.1	(0.8, 1.3)	1183	85	3.9*	(2.9, 5.0)	<.001
	RC	1	7	0.0	(0.0, 0.1)	8	10	0.1	(0.0, 0.2)	116	8	2.1*	(0.2, 3.9)	.028
	Other	5	33	0.1	(0.0, 0.1)	1	1	0.0	(0.0, 0.1)	98	7	0.9	(−3.8, 5.7)	.726
Adults (25–64)	All	160	100	0.7	(0.6, 0.8)	452	100	1.6	(1.4, 1.7)	7760	100	3.5*	(2.9, 4.1)	<.001
	MC	73	46	0.3	(0.2, 0.4)	369	82	1.3	(1.2, 1.4)	5603	72	5.3*	(4.7, 5.9)	<.001
	RC	24	15	0.1	(0.1, 0.1)	71	16	0.2	(0.2, 0.3)	1045	13	2.5	(−2.5, 7.8)	.328
	Other	63	39	0.3	(0.2, 0.4)	12	3	0.1	(0.0, 0.1)	1112	14	−3.9*	(−5.3, −2.4)	<.001
Older people (65–84)	All	220	100	3.2	(2.8, 3.6)	258	100	3.0	(2.6, 3.3)	6927	100	−0.7	(−1.9, 0.5)	.240
	MC	16	7	0.2	(0.1, 0.3)	138	53	1.6	(1.3, 1.9)	1859	27	7.3*	(5.6, 9.0)	<.001
	RC	51	23	0.8	(0.5, 1.0)	100	39	1.1	(0.9, 1.4)	2467	36	1.2	(−0.4, 2.8)	.145
	Other	153	70	2.2	(1.9, 2.6)	20	8	0.2	(0.1, 0.3)	2601	38	−10.1*	(−13.9,−6.1)	<.001
Elderly (85+)	All	31	100	3.9	(2.5, 5.3)	49	100	3.7	(2.6, 4.7)	1773	100	0.0	(−2.5, 2.5)	.992
	MC	2	6	0.3	(−0.1, 07)	3	6	0.4	(0.0, 0.7)	75	4	1.3	(−9.2, 12.9)	.820
	RC	11	35	1.2	(0.5, 2.0)	43	88	3.2	(2.3, 4.2)	846	48	5.5*	(3.2, 8.0)	<.001
	Other	18	58	2.2	(1.1, 3.2)	3	6	0.2	(0.0, 0.5)	852	48	−8.7*	(−13.9, −3.2)	0.002

**Abbreviations:** MC, microscopically confirmed; RC, radiographically confirmed; CNS, central nervous system.

* Indicates a statistically significant departure (*P* <.05) from a slope of 0.

a For ASRs standardized to the United States and World Standard Populations, please refer to Supplementary Table 4 where data for 2017 is presented and Supplementary Figure 1 for comparison purposes.

b Behavior based on the 5th digit of the ICD-0-3 histology code.

## Key Points

- The incidence rate of malignant central nervous system (CNS) tumors has remained stable in England during 1993–2017.
- Over the same period, the incidence rate of benign CNS tumors has increased.
- Most of the increase is among radiographically confirmed tumors and in people aged 65+ years.

## Importance of the Study

This is the first study to report temporal trends in the incidence of primary central nervous system (CNS) tumors for the entire population of England according to tumor behavior, method of diagnosis, and age. For all CNS tumors, there was a modest increase in the age-sex-standardized annual incidence rate over the 25-year study period (1993–2017). This was driven chiefly by a rapid increase in tumors that were radiographically rather than microscopically confirmed. Among radiographically confirmed tumors, the rate of increase was much greater for benign tumors than for malignant tumors, but for both groups, the majority of the increase was in people aged at least 65 years. These increases may be due in large part to an increase in the number of cases diagnosed incidentally by CT scans of the head conducted among older people attending accident and emergency departments, rather than to an increase in the underlying disease incidence rates.
