# Supplementary material for: Temporal trends in the incidence of malignant and nonmalignant primary brain and central nervous system tumors by the method of diagnosis in England, 1993–2017
Source: Neuro Oncol. Author manuscript; Available in PMC 2023 Jun 3. (PMC10237429; doi:10.1093/neuonc/noad001)
Supplement: Supplementary Material [file EMS170629-supplement-Supplementary_Material.pdf]

Supplementary Material

Title:

Temporal trends in the incidence of malignant and nonmalignant primary brain and central nervous system tumors by the method of diagnosis in England, 1993-2017

Running Title:

Temporal trends in CNS tumor incidence in England, 1993-2017

Usama M. Ali[1], Diana R. Withrow[2], Andrew D. Judge[3,4], Puneet Plaha[5,6], Sarah C. Darby[1]

1. Nuffield Department of Population Health, University of Oxford, Oxford, United Kingdom (UMA, SCD)
2. Nuffield Department of Primary Care Health Sciences, University of Oxford, Oxford, United Kingdom (DRW)
3. Nuffield Department of Orthopaedics, Rheumatology and Musculoskeletal Sciences, University of Oxford, Oxford, United Kingdom (ADJ)
4. Bristol NIHR Biomedical Research Centre and University of Bristol, Bristol, United Kingdom (ADJ)
5. Nuffield Department of Surgical Sciences, University of Oxford, Oxford, United Kingdom (PP)
6. Department of Neurosurgery, John Radcliffe Hospital, Oxford University Hospital NHS Foundation Trust, West Wing, Level 3, Oxford, United Kingdom (PP)

Corresponding author:

Usama M. Ali, MSc

Nuffield Department of Population Health, University of Oxford

Richard Doll Building, Roosevelt Drive, Oxford, OX3 7LF, United Kingdom

E-mail: usama.ali@wolfson.ox.ac.uk ; Telephone: +44 (0)1865 743849

ORCID iD: 0000-0003-0313-4429

Contents

Supplementary Table 1. Method of diagnosis criteria used by NCRAS as outlined in the International Classification of Diseases for Oncology Third Edition (ICD-O-3)

Supplementary Table 2. ICD-10 and ICD-9 Topography codes for tumors of the central nervous system (CNS)

Supplementary Table 3. Patient and tumor characteristics of 188,340 individuals diagnosed with a primary CNS tumor according to method of diagnosis – England, 1993-2017 *(column percentages)*

Supplementary Table 4. Comparison of Age-Sex-Standardized Incidence Rates (ASR) per 100,000 using different standard populations for 9,835 individuals diagnosed with a primary CNS tumor according to tumor behavior[a] and method of diagnosis – England, 2017 only

Supplementary Figure 1. Comparison of standard population weights – European (2013), United States (2000) and World (1960)

Supplementary Table 5a. Age-Sex-Standardized Incidence Rates (ASR) per 100,000 for calendar years 1993 and 2017 separately and Average Annual Percentage Change (AAPC) for 188,340 individuals diagnosed with a primary CNS tumor according to behavior, anatomical location[a] and method of diagnosis – England, 1993-2017 - Malignant brain tumors, benign meningeal tumors and all other tumors combined

Supplementary Table 5b. Age-Sex-Standardized Incidence Rates (ASR) per 100,000 for calendar years 1993 and 2017 separately and Average Annual Percentage Change (AAPC) for 188,340 individuals diagnosed with a primary CNS tumor according to behavior, anatomical location[a] and method of diagnosis – England, 1993-2017 - Malignant tumors only

Supplementary Table 5c. Age-Sex-Standardized Incidence Rates (ASR) per 100,000 for calendar years 1993 and 2017 separately and Average Annual Percentage Change (AAPC) for 188,340 individuals diagnosed with a primary CNS tumor according to behavior, anatomical location[a] and method of diagnosis – England, 1993-2017 - Benign tumors only

Supplementary Table 5d. Age-Sex-Standardized Incidence Rates (ASR) per 100,000 for calendar years 1993 and 2017 separately and Average Annual Percentage Change (AAPC) for 188,340 individuals diagnosed with a primary CNS tumor according to behavior, anatomical location[a] and method of diagnosis – England, 1993-2017 - Uncertain tumors only

Supplementary Figure 2. Incidence rate of primary CNS tumors by method of diagnosis, age-group and index of multiple deprivation (IMD) - England, 2001-2017

Supplementary Figure 3. Incidence rate of primary CNS tumors by anatomical location, method of diagnosis and age-group - England, 1993-2017 - Malignant tumors only

Supplementary Figure 4. Incidence of primary CNS tumors by anatomical location, method of diagnosis and age-group - England, 1993-2017 - Benign tumors only

Supplementary Figure 5. Incidence of primary CNS tumors by anatomical location, method of diagnosis and age-group - England, 1993-2017 - Uncertain tumors only

Supplementary Table 1. Method of diagnosis criteria used by NCRAS as outlined in the International Classification of Diseases for Oncology Third Edition (ICD-O-3)

Code	Description	Criteria	n	%	Category for this study
0	Death Certificate Only (DCO)	Information provided is from a death certificate	5,729	3	Other
1	Clinical	Diagnosis made before death, but without any of the following (codes 2-7).	12,435	7	Other
2	Clinical investigation	All diagnostic techniques, including x-ray, endoscopy, imaging, ultrasound, exploratory surgery (e.g., laparotomy), and autopsy, without a tissue diagnosis.	42,057	22	Radiographically Confirmed
3	Other special tests	Other special tests - no longer used	36	0	Other
4	Specific tumor markers	Including biochemical and/or immunological markers that are specific for a tumor site.	39	0	Other
5	Cytology	Examination of cells from a primary or secondary site, including fluids aspirated by endoscopy or needle; also includes the microscopic examination of peripheral blood and bone marrow aspirates.	268	0	Other
6	Histology of a metastasis	Histologic examination of tissue from a metastasis, including autopsy specimens	95	0	Other
7	Histology of a primary tumor	Histologic examination of tissue from primary tumor, however obtained, including all cutting techniques and bone marrow biopsies; also includes autopsy specimens of primary tumor.	124,190	66	Microscopically Confirmed
9	Unknown	No information on how the diagnosis has been made	3,491	2	Other

Note: Counts and percentages are for the entire study period 1993-2017

Supplementary Table 2. ICD-10 and ICD-9 Topography codes for tumors of the central nervous system (CNS)

Description	ICD-10 Code	ICD-9 Code
Malignant neoplasm of meninges	**C70**	**192**
Cerebral meninges	C70.0	192.1
Spinal meninges	C70.1	192.3
Meninges, unspecified	C70.9	192.9
Malignant neoplasm of brain	**C71**	**191**
Cerebrum, except lobes and ventricles	C71.0	191.0
Frontal lobe	C71.1	191.1
Temporal lobe	C71.2	191.2
Parietal lobe	C71.3	191.3
Occipital lobe	C71.4	191.4
Cerebral ventricle	C71.5	191.5
Cerebellum	C71.6	191.6
Brain stem	C71.7	191.7
Overlapping lesion of brain	C71.8	191.8
Brain, unspecified	C71.9	191.9
Malignant neoplasm of spinal cord, cranial nerves and other parts of central nervous system	**C72**	**192**
Spinal cord	C72.0	192.2
Cauda equina	C72.1	192.2
Olfactory nerve	C72.2	192.0
Optic nerve	C72.3	192.0
Acoustic nerve	C72.4	192.0
Other and unspecified cranial nerves	C72.5	192.0
Overlapping lesion of brain and other parts of CNS	C72.8	192.8
Central nervous system, unspecified	C72.9	192.9
Malignant neoplasm of other endocrine glands and related structures	**C75**	**194**
Pituitary gland	C75.1	194.3
Craniopharyngeal duct	C75.2	194.3
Pineal gland	C75.3	194.4
Benign neoplasm of meninges	**D32**	**225**
Cerebral meninges	D32.0	225.2
Spinal meninges	D32.1	225.4
Meninges, unspecified	D32.9	225.9
Benign neoplasm of brain and other parts of CNS	**D33**	**225**
Brain, supratentorial	D33.0	225.0
Brain, infratentorial	D33.1	225.0
Brain, unspecified	D33.2	225.9
Cranial nerves	D33.3	225.1
Spinal cord	D33.4	225.3
Other specified parts of the CNS	D33.7	225.8
Central nervous system, unspecified	D33.9	225.9
Benign neoplasm of other and unspecified endocrine glands	**D35**	**227**
Pituitary gland	D35.2	227.3
Craniopharyngeal duct	D35.3	227.3
Pineal gland	D35.4	227.4
Neoplasm of uncertain or unknown behavior of meninges	**D42**	**237**
Cerebral meninges	D42.0	237.6
Spinal meninges	D42.1	237.6
Meninges, unspecified	D42.9	237.9
Neoplasm of uncertain or unknown behavior of brain and CNS	**D43**	**237**
Brain, supratentorial	D43.0	237.5
Brain, infratentorial	D43.1	237.5
Brain, unspecified	D43.2	237.9
Cranial nerves	D43.3	237.9
Spinal cord	D43.4	237.5
Other parts of CNS	D43.7	237.9
Central nervous system, unspecified	D43.9	237.9
Neoplasm of uncertain or unknown behavior of endocrine glands	**D44**	**237**
Pituitary gland	D44.3	237.0
Craniopharyngeal duct	D44.4	237.0
Pineal gland	D44.5	237.1

Supplementary Table 3. Patient and tumor characteristics of 188,340 individuals diagnosed with a primary CNS tumor according to method of diagnosis – England, 1993-2017 *(column percentages)*

Characteristic	MC		RC		Other		Total	
	n	%	n	%	n	%	n	%
**Sex**								
Male	64,124	52	18,679	44	10,444	47	93,247	50
Female	60,066	48	23,378	56	11,649	53	95,093	50
**Age-group at diagnosis**								
Pediatric (0-14)	7,600	6	1,197	3	404	2	9,201	5
Teenagers & Young Adults (15-24)	4,890	4	642	2	393	2	5,925	3
Adults (25-64)	75,596	61	10,530	25	6,066	27	92,192	49
Older People (65-84)	35,252	28	22,843	54	11,847	54	69,942	37
Elderly (85+)	852	1	6,845	16	3,383	15	11,080	6
**Age at diagnosis**								
Median (IQR)	55	(41-66)	74	(61-82)	73	(60-81)	61	(46-73)
**Year of diagnosis**								
1993	3,569	3	558	1	1,444	7	5,571	3
1994	3,630	3	772	2	1,167	5	5,569	3
1995	3,949	3	835	2	1,305	6	6,089	3
1996	4,208	3	746	2	1,186	5	6,140	3
1997	4,369	4	836	2	1,244	6	6,449	3
1998	4,381	4	864	2	1,092	5	6,337	3
1999	4,283	3	849	2	1,383	6	6,515	3
2000	4,475	4	1,132	3	1,181	5	6,788	4
2001	4,478	4	1,195	3	952	4	6,625	4
2002	4,646	4	1,289	3	769	3	6,704	4
2003	4,569	4	1,328	3	752	3	6,649	4
2004	4,841	4	1,494	4	647	3	6,982	4
2005	5,061	4	1,422	3	808	4	7,291	4
2006	5,091	4	1,474	4	739	3	7,304	4
2007	5,407	4	1,608	4	659	3	7,674	4
2008	5,645	5	1,701	4	864	4	8,210	4
2009	5,284	4	1,908	5	865	4	8,057	4
2010	5,304	4	1,988	5	805	4	8,097	4
2011	5,367	4	2,023	5	756	3	8,146	4
2012	5,312	4	2,307	5	921	4	8,540	5
2013	6,081	5	2,511	6	1,009	5	9,601	5
2014	5,951	5	3,031	7	564	3	9,546	5
2015	6,102	5	3,289	8	462	2	9,853	5
2016	6,063	5	3,433	8	272	1	9,768	5
2017	6,124	5	3,464	8	247	1	9,835	5
**Tumor behavior[a]**								
Malignant	66,684	54	20,280	48	11,067	50	98,031	52
Benign	46,033	37	17,076	41	6,249	28	69,358	37
Uncertain	11,473	9	4,701	11	4,777	22	20,951	11
**Anatomical location[b]**								
Meninges (C70.0-C70.9, D32.0-D32.9, D42.0-D42.9)	26,604	21	11,518	27	4,121	19	42,243	22
Brain (C71.0-C71.9, D33.0-D33.2, D43.0-D43.2)	70,620	57	23,447	56	14,325	65	108,463	58
Spinal Cord & Other CNS (C72.0-C72.9, D33.3-D33.9, D43.3-D43.9)	12,318	10	3,466	8	1,545	7	17,329	9
Endocrine CNS (C75.1-C75.3, D35.2-D35.4, D44.3-D44.5)	14,648	12	3,626	9	2,102	10	20,305	11
**Anatomical location according to behavior**								
Malignant								
Meninges	1,129	1	332	1	276	1	1,737	1
Brain	62,760	51	19,224	46	10,266	46	92,250	49
Spinal Cord & Other CNS	1,948	2	552	1	295	1	2,795	1
Endocrine Glands in CNS	847	1	172	0	230	1	1,249	1
Benign								
Meninges	23,194	19	10,936	26	3,676	17	37,806	20
Brain	1,842	1	559	1	335	2	2,736	1
Spinal Cord & Other CNS	8,996	7	2,652	6	1,012	5	12,660	7
Endocrine Glands in CNS	12,001	10	2,929	7	1,226	6	16,156	9
Uncertain								
Meninges	2,281	2	250	1	169	1	2,700	1
Brain	6,018	5	3,664	9	3,724	17	13,406	7
Spinal Cord & Other CNS	1,374	1	262	1	238	1	1,874	1
Endocrine Glands in CNS	1,800	1	525	1	646	3	2,971	2
**ICD-10 Site**								
Cerebral meninges (C70.0)	815	1	242	1	166	1	1,223	1
Spinal meninges (C70.1)	110	0	13	0	13	0	136	0
Meninges, unspecified (C70.9)	286	0	112	0	121	1	519	0
Cerebrum, except lobes and ventricles (C71.0)	3,183	3	1,387	3	987	4	5,557	3
Frontal lobe (C71.1)	16,202	13	2,940	7	904	4	20,046	11
Temporal lobe (C71.2)	12,432	10	1,996	5	584	3	15,012	8
Parietal lobe (C71.3)	10,340	8	2,098	5	691	3	13,129	7
Occipital lobe (C71.4)	2,485	2	398	1	158	1	3,041	2
Cerebral ventricle (C71.5)	697	1	115	0	56	0	868	0
Cerebellum (C71.6)	3,565	3	261	1	212	1	4,038	2
Brain stem (C71.7)	1,196	1	655	2	235	1	2,086	1
Overlapping lesion of brain (C71.8)	3,732	3	1,415	3	366	2	5,513	3
Brain, unspecified (C71.9)	11,454	9	8,016	19	6,130	28	25,600	14
Spinal cord (C72.0)	1,602	1	159	0	138	1	1,899	1
Cauda equina (C72.1)	18	0	2	0	2	0	22	0
Olfactory nerve (C72.2)	46	0	2	0	8	0	56	0

Continued overleaf

Supplementary Table 3 continued.

Characteristic	MC		RC		Other		Total	
	n	%	n	%	n	%	n	%
Optic nerve (C72.3)	282	0	347	1	66	0	695	0
Acoustic nerve (C72.4)	36	0	9	0	11	0	56	0
Other and unspecified cranial nerves (C72.5)	157	0	41	0	34	0	232	0
Overlapping lesion of brain and other parts of CNS (C72.8)	15	0	4	0	7	0	26	0
Central nervous system, unspecified (C72.9)	73	0	21	0	37	0	131	0
Pituitary gland (C75.1)	292	0	128	0	195	1	615	0
Craniopharyngeal duct (C75.2)	4	0	1	0	5	0	10	0
Pineal gland (C75.3)	628	1	64	0	51	0	743	0
Cerebral meninges (D32.0)	17,187	14	8,483	20	2,253	10	27,923	15
Spinal meninges (D32.1)	1,988	2	140	0	76	0	2,204	1
Meninges, unspecified (D32.9)	3,938	3	2,278	5	1,324	6	7,540	4
Brain, supratentorial (D33.0)	889	1	176	0	71	0	1,136	1
Brain, infratentorial (D33.1)	262	0	42	0	31	0	335	0
Brain, unspecified (D33.2)	801	1	345	1	229	1	1,375	1
Cranial nerves (D33.3)	7,131	6	2,496	6	937	4	10,564	6
Spinal cord (D33.4)	1,580	1	114	0	43	0	1,737	1
Other specified parts of the CNS (D33.7)	34	0	0	0	5	0	39	0
Central nervous system, unspecified (D33.9)	216	0	33	0	21	0	270	0
Pituitary gland (D35.2)	11,911	10	2,899	7	1,207	5	16,017	9
Craniopharyngeal duct (D35.3)	4	0	1	0	1	0	6	0
Pineal gland (D35.4)	20	0	13	0	2	0	35	0
Cerebral meninges (D42.0)	1,835	1	173	0	84	0	2,092	1
Spinal meninges (D42.1)	81	0	8	0	11	0	100	0
Meninges, unspecified (D42.9)	364	0	69	0	73	0	506	0
Brain, supratentorial (D43.0)	1,103	1	1,216	3	708	3	3,027	2
Brain, infratentorial (D43.1)	864	1	212	1	203	1	1,279	1
Brain, unspecified (D43.2)	1,415	1	2,175	5	2,760	12	6,350	3
Cranial nerves (D43.3)	56	0	79	0	53	0	188	0
Spinal cord (D43.4)	945	1	111	0	76	0	1,132	1
Other parts of CNS (D43.7)	2	0	1	0	7	0	10	0
Central nervous system, unspecified (D43.9)	125	0	47	0	100	0	272	0
Pituitary gland (D44.3)	490	0	291	1	480	2	1,261	1
Craniopharyngeal duct (D44.4)	1,115	1	158	0	127	1	1,400	1
Pineal gland (D44.5)	184	0	71	0	34	0	289	0
**Total (all CNS tumors)**	124,553	100	54,567	100	5,729	100	188,340	100

Abbreviations: MC, microscopically confirmed; RC, radiographically confirmed; CNS, central nervous system.

[a] Behavior based on the 5th digit of the ICD-O-3 histology code.

[b] Anatomical location based on the ICD-10 topography code.

Supplementary Table 4. Comparison of Age-Sex-Standardized Incidence Rates (ASR) per 100,000 using different standard populations for 9,835 individuals diagnosed with a primary CNS tumor according to tumor behavior[a] and method of diagnosis – England, 2017 only

Characteristic	Method of Diagnosis	n	%	European (2013) ASR	European (2013) 95% CI	United States (2000) ASR	United States (2000) 95% CI	World (1960) ASR	World (1960) 95% CI
**All CNS Tumors**									
All ages	All	9,835	100	18.6	(18.2,18.9)	15.1	(14.8,15.4)	11.9	(11.6,12.1)
	MC	6,124	62	11.5	(11.2,11.8)	9.8	(9.6,10.1)	8.5	(8.2,8.7)
	RC	3,464	35	6.6	(6.4,6.8)	4.9	(4.7,5.1)	3.2	(3.0,3.3)
	Other	247	3	0.5	(0.4,0.5)	0.4	(0.3,0.4)	0.2	(0.2,0.3)
**All CNS Tumors**									
Pediatric (0-14)	All	422	100	4.2	(3.8,4.6)	4.2	(3.8,4.6)	4.2	(3.8,4.6)
	MC	341	81	3.4	(3.0,3.7)	3.4	(3.0,3.7)	3.4	(3.1,3.8)
	RC	69	16	0.7	(0.5,0.8)	0.7	(0.5,0.8)	0.7	(0.5,0.8)
	Other	12	3	0.1	(0.1,0.2)	0.1	(0.1,0.2)	0.1	(0.1,0.2)
Teenagers & Young Adults (15-24)	All	298	100	4.5	(4.0,5.0)	4.4	(3.9,4.9)	4.4	(3.9,4.9)
	MC	235	79	3.5	(3.1,4.0)	3.5	(3.0,3.9)	3.5	(3.0,3.9)
	RC	59	20	0.9	(0.7,1.1)	0.9	(0.6,1.1)	0.9	(0.6,1.1)
	Other	4	1	0.1	(0.0,0.2)	0.1	(0.0,0.1)	0.1	(0.0,0.1)
Adults (25-64)	All	4,688	100	16.6	(16.1,17.0)	15.3	(14.8,15.7)	15.1	(14.6,15.5)
	MC	3,598	77	12.7	(12.3,13.1)	11.8	(11.4,12.2)	11.6	(11.2,12.0)
	RC	1,025	22	3.6	(3.4,3.9)	3.3	(3.1,3.5)	3.2	(3.0,3.5)
	Other	65	1	0.2	(0.2,0.3)	0.2	(0.2,0.3)	0.2	(0.2,0.3)
Older People (65-84)	All	3,783	100	43.7	(42.3,45.1)	43.9	(42.5,45.3)	42.6	(41.1,44.0)
	MC	1,911	51	22.0	(21.0,23.0)	21.7	(20.8,22.7)	23.9	(22.8,25.0)
	RC	1,748	46	20.2	(19.3,21.2)	20.7	(19.7,21.6)	17.4	(16.6,18.3)
	Other	124	3	1.4	(1.2,1.7)	1.5	(1.2,1.7)	1.2	(1.0,1.5)
Elderly (85+)	All	644	100	47.5	(43.8,51.2)	47.6	(44.0,51.3)	47.6	(44.0,51.3)
	MC	39	6	2.8	(1.9,3.7)	2.9	(2.0,3.8)	2.9	(2.0,3.8)
	RC	563	87	41.5	(38.1,45.0)	41.6	(38.2,45.1)	41.6	(38.2,45.1)
	Other	42	7	3.2	(2.2,4.1)	3.1	(2.2,4.0)	3.1	(2.2,4.0)
**Malignant behavior**									
Pediatric (0-14)	All	223	100	2.2	(1.9,2.5)	2.2	(1.9,2.5)	2.3	(2.0,2.6)
	MC	182	82	1.8	(1.5,2.0)	1.8	(1.5,2.0)	1.9	(1.6,2.1)
	RC	36	16	0.4	(0.2,0.5)	0.4	(0.2,0.5)	0.4	(0.2,0.5)
	Other	5	2	0.1	(0.0,0.3)	0.1	(0.0,0.3)	0.1	(0.0,0.3)
Teenagers & Young Adults (15-24)	All	123	100	1.8	(1.5,2.2)	1.8	(1.5,2.2)	1.8	(1.5,2.1)
	MC	108	88	1.6	(1.3,1.9)	1.6	(1.5,2.0)	1.6	(1.3,1.9)
	RC	13	11	0.2	(0.1,0.3)	0.2	(0.2,0.5)	0.2	(0.1,0.3)
	Other	2	2	0.0	(0.0,0.1)	0.0	(0.0,0.3)	0.0	(0.0,0.1)
Adults (25-64)	All	1,973	100	7.0	(6.7,7.3)	6.3	(6.0,6.6)	6.3	(6.0,6.6)
	MC	1,767	90	6.2	(5.9,6.5)	5.7	(1.5,2.0)	5.6	(5.4,5.9)
	RC	187	9	0.7	(0.6,0.8)	0.6	(0.2,0.5)	0.6	(0.5,0.7)
	Other	19	1	0.1	(0.1,0.1)	0.1	(0.0,0.3)	0.1	(0.0,0.1)
Older People (65-84)	All	1,966	100	22.7	(21.7,23.7)	22.8	(21.8,23.8)	22.1	(21.1,23.1)
	MC	1,105	56	12.7	(12.0,13.5)	12.5	(1.5,2.0)	14.0	(13.2,14.9)
	RC	798	41	9.3	(8.6,9.9)	9.5	(0.2,0.5)	7.4	(6.9,8.0)
	Other	63	3	0.7	(0.5,0.9)	0.7	(0.0,0.3)	0.6	(0.5,0.8)
Elderly (85+)	All	283	100	20.9	(18.4,23.3)	20.9	(18.5,23.4)	20.9	(18.5,23.4)
	MC	14	5	1.0	(0.5,1.6)	1.0	(1.5,2.0)	1.0	(0.5,1.6)
	RC	251	89	18.5	(16.2,20.8)	18.6	(0.2,0.5)	18.6	(16.3,20.9)
	Other	18	6	1.3	(0.7,2.0)	1.3	(0.0,0.3)	1.3	(0.7,1.9)
**Benign behavior**									
Pediatric (0-14)	All	37	100	0.4	(0.3,0.5)	0.4	(0.3,0.5)	0.4	(0.2,0.5)
	MC	25	68	0.3	(0.2,0.4)	0.3	(1.5,2.0)	0.2	(0.1,0.3)
	RC	10	27	0.1	(0.1,0.2)	0.1	(0.2,0.5)	0.2	(0.1,0.2)
	Other	2	5	0.0	(0.0,0.1)	0.0	(0.0,0.3)	0.0	(0.0,0.1)
Teenagers & Young Adults (15-24)	All	95	100	1.4	(1.1,1.7)	1.4	(1.1,1.7)	1.4	(1.1,1.7)
	MC	56	59	0.8	(0.6,1.1)	0.8	(1.5,2.0)	0.8	(0.6,1.0)
	RC	38	40	0.6	(0.4,0.8)	0.6	(0.2,0.5)	0.6	(0.4,0.7)
	Other	1	1	0.0	(0.0,0.1)	0.0	(0.0,0.3)	0.0	(0.0,0.1)
Adults (25-64)	All	2,263	100	8.0	(7.7,8.3)	7.5	(7.1,7.8)	7.3	(7.0,7.6)
	MC	1,462	65	5.2	(4.9,5.4)	4.9	(1.5,2.0)	4.7	(4.5,5.0)
	RC	767	34	2.7	(2.5,2.9)	2.5	(0.2,0.5)	2.4	(2.2,2.6)
	Other	34	2	0.1	(0.1,0.2)	0.1	(0.0,0.3)	0.1	(0.1,0.2)
Older People (65-84)	All	1,559	100	18.0	(17.1,18.9)	18.1	(17.2,19.0)	17.6	(16.7,18.5)
	MC	668	43	7.7	(7.1,8.3)	7.6	(1.5,2.0)	8.1	(7.5,8.8)
	RC	850	55	9.8	(9.2,10.5)	9.9	(0.2,0.5)	9.1	(8.4,9.7)
	Other	41	3	0.5	(0.3,0.6)	0.5	(0.0,0.3)	0.4	(0.3,0.5)
Elderly (85+)	All	312	100	23.0	(20.4,25.5)	23.1	(20.5,25.6)	23.1	(20.5,25.6)
	MC	22	7	1.6	(0.9,2.3)	1.6	(1.5,2.0)	1.6	(0.9,2.3)
	RC	269	86	19.8	(17.4,22.2)	19.9	(0.2,0.5)	19.9	(17.5,22.3)
	Other	21	7	1.6	(0.9,2.3)	1.6	(0.0,0.3)	1.6	(0.9,2.2)

Continued overleaf

Supplementary Table 4 continued.

Characteristic	Method of Diagnosis	n	%	European (2013)		United States (2000)		World (1960)	
				**ASR**	**95% CI**	**ASR**	**95% CI**	**ASR**	**95% CI**
**Uncertain behavior**									
Pediatric (0-14)	All	162	100	1.6	(1.4,1.9)	1.6	(1.4,1.9)	1.6	(1.4,1.9)
	MC	134	83	1.3	(1.1,1.6)	1.3	(1.5,2.0)	1.3	(1.1,1.5)
	RC	23	14	0.2	(0.1,0.3)	0.2	(0.2,0.5)	0.2	(0.1,0.3)
	Other	5	3	0.0	(0.0,0.1)	0.0	(0.0,0.3)	0.1	(0.0,0.1)
Teenagers & Young Adults (15-24)	All	80	100	1.2	(0.9,1.5)	1.2	(0.9,1.5)	1.2	(0.9,1.5)
	MC	71	89	1.1	(0.8,1.3)	1.1	(1.5,2.0)	1.1	(0.8,1.3)
	RC	8	10	0.1	(0.0,0.2)	0.1	(0.2,0.5)	0.1	(0.0,0.2)
	Other	1	1	0.0	(0.0,0.1)	0.0	(0.0,0.3)	0.0	(0.0,0.1)
Adults (25-64)	All	452	100	1.6	(1.4,1.7)	1.5	(1.4,1.7)	1.5	(1.4,1.7)
	MC	369	82	1.3	(1.2,1.4)	1.2	(1.5,2.0)	1.2	(1.1,1.4)
	RC	71	16	0.2	(0.2,0.3)	0.2	(0.2,0.5)	0.2	(0.2,0.3)
	Other	12	3	0.1	(0.0,0.1)	0.1	(0.0,0.3)	0.1	(0.0,0.1)
Older People (65-84)	All	258	100	3.0	(2.6,3.3)	3.0	(2.6,3.3)	2.9	(2.5,3.2)
	MC	138	53	1.6	(1.3,1.9)	1.6	(1.5,2.0)	1.7	(1.4,2.0)
	RC	100	39	1.1	(0.9,1.4)	1.2	(0.2,0.5)	0.9	(0.7,1.1)
	Other	20	8	0.2	(0.1,0.3)	0.2	(0.0,0.3)	0.2	(0.1,0.3)
Elderly (85+)	All	49	100	3.7	(2.6,4.7)	3.6	(2.6,4.6)	3.6	(2.6,4.6)
	MC	3	6	0.4	(0.0,0.7)	0.2	(1.5,2.0)	0.2	(0.0,0.5)
	RC	43	88	3.2	(2.3,4.2)	3.2	(0.2,0.5)	3.2	(2.2,4.1)
	Other	3	6	0.2	(0.0,0.5)	0.2	(0.0,0.3)	0.2	(0.0,0.5)

Abbreviations: MC, microscopically confirmed; RC, radiographically confirmed; CNS, central nervous system.

[a] Behavior based on the 5th digit of the ICD-O-3 histology code.

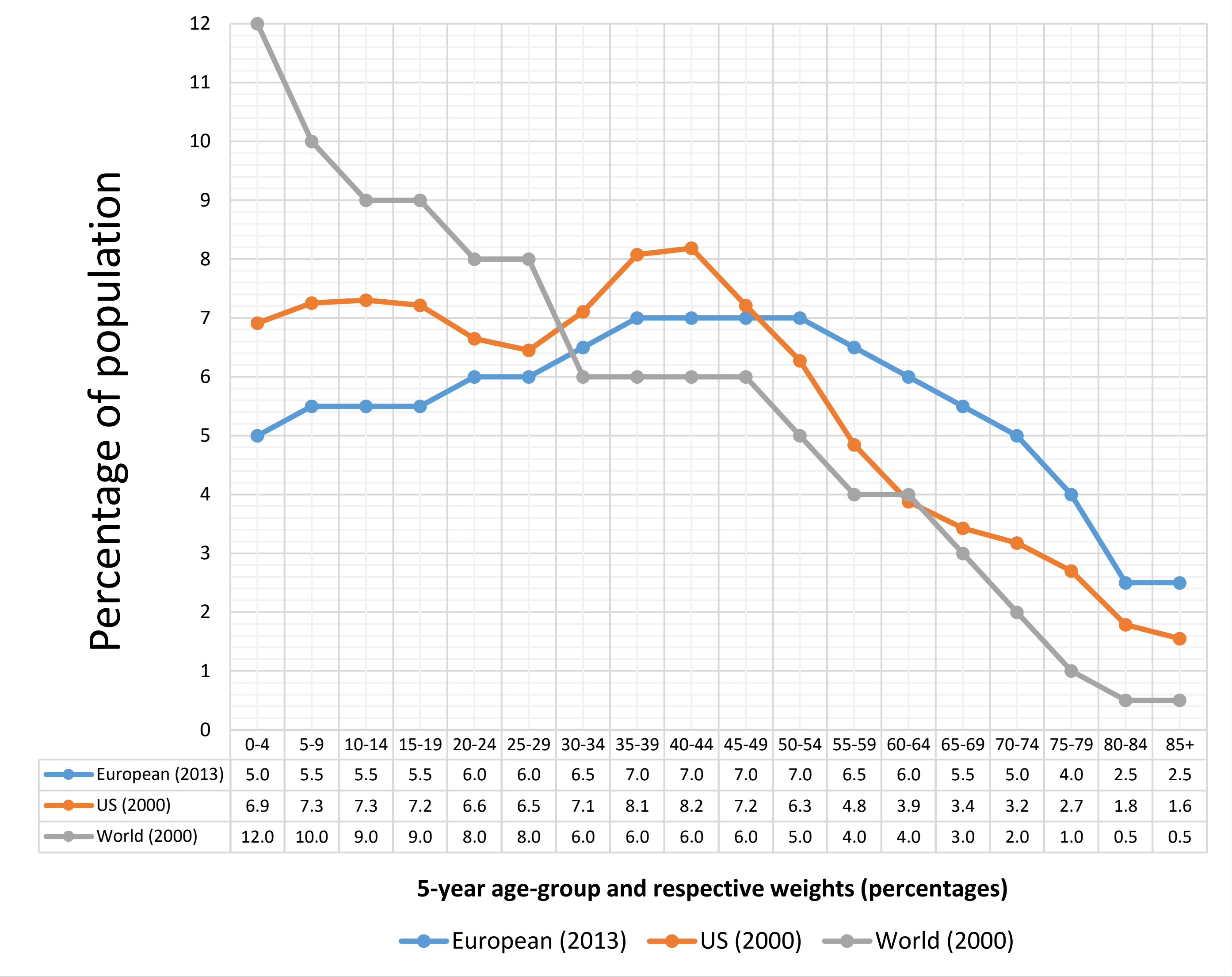

	0-4	5-9	10-14	15-19	20-24	25-29	30-34	35-39	40-44	45-49	50-54	55-59	60-64	65-69	70-74	75-79	80-84	85+
European (2013)	5.0	5.5	5.5	5.5	6.0	6.0	6.5	7.0	7.0	7.0	7.0	6.5	6.0	5.5	5.0	4.0	2.5	2.5
US (2000)	6.9	7.3	7.3	7.2	6.6	6.5	7.1	8.1	8.2	7.2	6.3	4.8	3.9	3.4	3.2	2.7	1.8	1.6
World (2000)	12.0	10.0	9.0	9.0	8.0	8.0	6.0	6.0	6.0	6.0	5.0	4.0	4.0	3.0	2.0	1.0	0.5	0.5

Supplementary Figure 1. Comparison of standard population weights – European (2013), United States (2000) and World (1960)

Supplementary Table 5a. Age-Sex-Standardized Incidence Rates (ASR) per 100,000 for calendar years 1993 and 2017 separately and Average Annual Percentage Change (AAPC) for 188,340 individuals diagnosed with a primary CNS tumor according to behavior and anatomical location[a] and method of diagnosis – England, 1993-2017 - Malignant brain tumors, benign meningeal tumors and all other tumors combined

Characteristic	Method of Diagnosis	1993 only				2017 only				1993-2017				
		n	%	ASR	95% CI	n	%	ASR	95% CI	n	%	AAPC	95% CI	P-Value
**Malignant behavior - Brain**														
Pediatric (0-14)	All	213	100	2.3	(2.0,2.6)	254	100	2.5	(2.2,2.8)	4,338	100	-0.1	(-0.5,0.3)	0.652
	MC	176	83	1.9	(1.6,2.2)	223	88	2.2	(1.9,2.5)	3,607	83	-0.3	(-0.7,0.1)	0.178
	RC	11	5	0.1	(0.0,0.2)	27	11	0.3	(0.2,0.4)	555	13	3.6*	(2.1,5.2)	< 0.001
	Other	26	12	0.3	(0.2,0.4)	4	2	0.1	(0.0,0.1)	176	4	-6.5*	(-8.7,-4.3)	< 0.001
Teenagers & Young Adults (15-24)	All	113	100	1.8	(1.5,2.1)	124	100	1.9	(1.5,2.2)	2,408	100	0.1	(-0.5,0.7)	0.825
	MC	85	75	1.3	(1.1,1.6)	114	92	1.7	(1.4,2.0)	2,091	87	0.2	(-0.3,0.8)	0.417
	RC	2	2	0.0	(0.0,0.1)	9	7	0.1	(0.0,0.2)	178	7	2.3	(-0.4,5.1)	0.094
	Other	26	23	0.4	(0.3,0.6)	1	1	0.0	(0.0,0.1)	139	6	-6.0*	(-8.8,-3.1)	< 0.001
Adults (25-64)	All	1,658	100	7.3	(6.9,7.6)	1,887	100	6.7	(6.4,7.0)	43,649	100	-0.3	(-1.4,0.7)	0.53
	MC	1,238	75	5.4	(5.1,5.7)	1,699	90	6.0	(5.7,6.3)	37,453	86	0.4	(-0.2,1.0)	0.153
	RC	105	6	0.5	(0.4,0.6)	175	9	0.6	(0.5,0.7)	3,467	8	1.4*	(0.5,2.4)	0.005
	Other	315	19	1.4	(1.3,1.6)	13	1	0.1	(0.0,0.1)	2,729	6	-9.0*	(-10.1,-7.9)	< 0.001
Older People (65-84)	All	1,125	100	16.4	(15.4,17.4)	1,928	100	22.3	(21.3,23.3)	37,721	100	1.4*	(0.8,2.0)	< 0.001
	MC	477	42	6.9	(6.3,7.5)	1,075	56	12.4	(11.6,13.1)	19,406	51	2.5*	(1.9,3.2)	< 0.001
	RC	215	19	3.1	(2.7,3.6)	791	41	9.2	(8.5,9.8)	12,222	32	4.0*	(3.0,5.1)	< 0.001
	Other	433	38	6.4	(5.8,7.0)	62	3	0.7	(0.5,0.9)	6,093	16	-9.5*	(-12.8,-6.1)	< 0.001
Elderly (85+)	All	54	100	6.2	(4.5,7.8)	281	100	20.7	(18.3,23.1)	4,134	100	5.3*	(3.9,6.7)	< 0.001
	MC	2	4	0.3	(-0.1,0.7)	13	5	1.0	(0.4,1.5)	203	5	-0.5	(-2.7,1.7)	0.636
	RC	17	31	1.9	(1.0,2.8)	250	89	18.4	(16.1,20.7)	2,802	68	9.5*	(7.6,11.5)	< 0.001
	Other	35	65	4.0	(2.7,5.4)	18	6	1.3	(0.7,2.0)	1,129	27	-4.5*	(-8.2,-0.6)	0.025
**Benign behavior - Meninges**														
Pediatric (0-14)	All	2	100	0.1	(0.0,0.2)	2	100	0.1	(0.0,0.2)	83	100	-0.7	(-2.9,1.6)	0.534
	MC	1	50	0.0	(0.0,0.1)	1	50	0.0	(0.0,0.1)	69	83	-1.7	(-4.1,0.7)	0.16
	RC	0	0	–	–	1	50	0.0	(0.0,0.1)	9	11	-0.4	(-1.2,0.4)	0.264
	Other	1	50	0.0	(0.0,0.1)	0	0	–	–	5	6	-0.1	(-0.4,0.2)	0.446
Teenagers & Young Adults (15-24)	All	12	100	0.2	(0.1,0.3)	13	100	0.2	(0.1,0.3)	271	100	0.5	(-1.0,2.1)	0.479
	MC	11	92	0.2	(0.1,0.3)	10	77	0.1	(0.1,0.2)	230	85	-0.5	(-2.2,1.3)	0.597
	RC	0	0	–	–	3	23	0.0	(0.0,0.1)	30	11	1.2	(-3.1,5.7)	0.568
	Other	1	8	0.0	(0.0,0.1)	0	0	–	–	11	4	-0.4	(-5.7,5.3)	0.878
Adults (25-64)	All	383	100	1.7	(1.5,1.9)	1,148	100	4.1	(3.9,4.3)	17,849	100	3.0*	(0.8,5.3)	0.006
	MC	327	85	1.5	(1.3,1.6)	745	65	2.7	(2.5,2.8)	14,583	82	2.1*	(0.1,4.1)	0.039
	RC	11	3	0.1	(0.0,0.2)	389	34	1.4	(1.3,1.5)	2,585	14	9.9*	(7.7,12.1)	< 0.001
	Other	45	12	0.2	(0.1,0.3)	14	1	0.1	(0.0,0.1)	681	4	-5.7	(-13.0,2.3)	0.16
Older People (65-84)	All	329	100	4.8	(4.3,5.3)	1,053	100	12.2	(11.5,12.9)	15,425	100	3.0*	(0.8,5.3)	0.006
	MC	217	66	3.2	(2.7,3.6)	421	40	4.9	(4.4,5.3)	7,925	51	1.5*	(1.0,2.0)	< 0.001
	RC	29	9	0.4	(0.3,0.6)	598	57	6.9	(6.4,7.5)	5,609	36	9.0*	(6.5,11.5)	< 0.001
	Other	83	25	1.2	(0.9,1.5)	34	3	0.4	(0.3,0.5)	1,891	12	-5.3*	(-9.7,-0.7)	0.025
Elderly (85+)	All	45	100	5.6	(3.9,7.2)	251	100	18.6	(16.3,20.9)	4,178	100	4.8*	(3.4,6.2)	< 0.001
	MC	12	27	1.5	(0.6,2.3)	15	6	1.1	(0.5,1.7)	387	9	0.3	(-1.2,1.8)	0.728
	RC	7	16	0.8	(0.2,1.4)	218	87	16.1	(14.0,18.2)	2,703	65	9.3*	(7.7,10.8)	< 0.001
	Other	26	58	3.3	(2.0,4.6)	18	7	1.4	(0.7,2.0)	1,088	26	-4.6	(-9.1,0.1)	0.057
**All Other CNS Tumors**														
Pediatric (0-14)	All	100	100	1.1	(0.9,1.3)	166	100	1.7	(1.4,1.9)	4,780	100	1.9*	(1.4,2.4)	< 0.001
	MC	75	75	0.8	(0.6,1.0)	117	70	1.2	(1.0,1.4)	3,924	82	1.6*	(1.0,2.3)	< 0.001
	RC	7	7	0.1	(0.0,0.1)	41	25	0.4	(0.3,0.5)	633	13	6.8*	(3.7,9.9)	< 0.001
	Other	18	18	0.2	(0.1,0.3)	8	5	0.1	(0.0,0.1)	223	5	-3.6*	(-5.6,-1.6)	0.001
Teenagers & Young Adults (15-24)	All	68	100	1.1	(0.8,1.3)	161	100	2.4	(2.0,2.8)	3,246	100	2.4*	(1.7,3.0)	< 0.001
	MC	44	65	0.7	(0.5,0.9)	111	69	1.7	(1.4,2.0)	2,569	79	2.1*	(1.3,2.9)	< 0.001
	RC	2	3	0.0	(0.0,0.1)	47	29	0.7	(0.5,0.9)	434	13	7.7*	(5.7,9.6)	< 0.001
	Other	22	32	0.3	(0.2,0.5)	3	2	0.0	(0.0,0.1)	243	7	-3.2*	(-5.6,-0.8)	0.012
Adults (25-64)	All	895	100	3.8	(3.6,4.1)	1,653	100	5.8	(5.5,6.1)	30,694	100	1.8	(-0.1,3.8)	0.069
	MC	682	76	2.9	(2.7,3.1)	1,154	70	4.0	(3.8,4.3)	23,560	77	1.5*	(1.1,2.0)	< 0.001
	RC	51	6	0.2	(0.2,0.3)	461	28	1.6	(1.5,1.8)	4,478	15	8.6*	(5.4,11.8)	< 0.001
	Other	162	18	0.7	(0.6,0.8)	38	2	0.1	(0.1,0.2)	2,656	9	-4.3*	(-6.0,-2.5)	< 0.001
Older People (65-84)	All	529	100	7.7	(7.1,8.4)	802	100	9.2	(8.6,9.9)	16,796	100	1.4*	(1.0,1.7)	< 0.001
	MC	215	41	3.1	(2.7,3.5)	415	52	4.8	(4.3,5.2)	7,921	47	2.5*	(0.4,4.6)	0.018
	RC	87	16	1.3	(1.0,1.5)	359	45	4.1	(3.7,4.6)	5,012	30	4.5*	(3.8,5.2)	< 0.001
	Other	227	43	3.3	(2.9,3.7)	28	3	0.3	(0.2,0.4)	3,863	23	-9.2*	(-12.8,-5.6)	< 0.001
Elderly (85+)	All	45	100	5.4	(3.8,7.0)	112	100	8.2	(6.7,9.8)	2,768	100	0.6	(-0.9,2.0)	0.45
	MC	7	16	0.9	(0.2,1.5)	11	10	0.8	(0.3,1.2)	262	9	0.4	(-1.8,2.7)	0.69
	RC	14	31	1.6	(0.8,2.5)	95	85	7.0	(5.6,8.4)	1,340	48	7.1*	(5.0,9.2)	< 0.001
	Other	24	53	2.9	(1.7,4.1)	6	5	0.5	(0.1,0.8)	1,166	42	-8.0*	(-12.4,-3.4)	0.001

Abbreviations: MC, microscopically confirmed; RC, radiographically confirmed; CNS, central nervous system.
*Indicates a statistically significant departure (p<0.05) from a slope of 0.
[a]Anatomical location based on the ICD-10 topography codes.

Supplementary Table 5b. Age-Sex-Standardized Incidence Rates (ASR) per 100,000 for calendar years 1993 and 2017 separately and Average Annual Percentage Change (AAPC) for 188,340 individuals diagnosed with a primary CNS tumor according to behavior, anatomical location[a] and method of diagnosis – England, 1993-2017 - Malignant tumors only

Characteristic	Method of Diagnosis	1993 only				2017 only				1993-2017				
		n	%	ASR	95% CI	n	%	ASR	95% CI	n	%	AAPC	95% CI	P-Value
**Malignant behavior - Meninges**														
Pediatric (0-14)	All	3	100	0.0	(0.0,0.1)	1	100	0.0	(0.0,0.1)	34	100	-0.5	(-1.9,1.0)	0.482
	MC	3	100	0.0	(0.0,0.1)	1	100	0.0	(0.0,0.1)	33	97	-0.8	(-2.0,0.4)	0.157
	RC	–	–	–	–	–	–	–	–	1	3	–	–	–
	Other	–	–	–	–	–	–	–	–	–	–	–	–	–
Teenagers & Young Adults (15-24)	All	1	100	0.0	(0.0,0.1)	1	100	0.0	(0.0,0.1)	23	100	-0.7	(-3.4,2.0)	0.57
	MC	–	–	–	–	1	100	0.0	(0.0,0.1)	20	87	0.1	(-2.8,3.0)	0.967
	RC	–	–	–	–	–	–	–	–	2	9	-1.4	–	–
	Other	1	100	0.0	(0.0,0.1)	–	–	–	–	1	4	–	–	–
Adults (25-64)	All	58	100	0.3	(0.2,0.4)	25	100	0.1	(0.1,0.2)	737	100	0.0	(-1.4,1.5)	0.959
	MC	46	79	0.2	(0.2,0.3)	25	100	0.1	(0.1,0.2)	639	87	0.4	(-0.9,1.7)	0.572
	RC	6	10	0.1	(0.0,0.1)	–	–	–	–	42	6	-1.7	(-3.5,0.2)	0.07
	Other	6	10	0.1	(0.0,0.1)	–	–	–	–	56	8	2.0	(-2.8,7.0)	0.427
Older People (65-84)	All	54	100	0.8	(0.6,1.0)	16	100	0.2	(0.1,0.3)	764	100	-4.1	(-8.1,0.0)	0.053
	MC	29	54	0.4	(0.3,0.6)	15	94	0.2	(0.1,0.3)	416	54	-0.4	(-1.9,1.2)	0.625
	RC	13	24	0.2	(0.1,0.3)	1	6	0.1	(-0.1,0.2)	195	26	1.3	(-1.4,4.1)	0.347
	Other	12	22	0.2	(0.1,0.3)	–	–	–	–	153	20	-3.5*	(-6.7,-0.2)	0.04
Elderly (85+)	All	2	100	0.4	(-0.1,0.8)	1	100	0.2	(-0.2,0.6)	179	100	-7.9	(-15.9,1.0)	0.079
	MC	–	–	–	–	1	100	0.2	(-0.2,0.6)	21	12	-2.9*	(-5.4,-0.2)	0.035
	RC	1	50	0.2	(-0.2,0.5)	–	–	–	–	92	51	2.4	(-2.4,7.5)	0.31
	Other	1	50	0.2	(-0.2,0.5)	–	–	–	–	66	37	0.8	(-2.7,4.5)	0.632
**Malignant behavior - Brain**														
Pediatric (0-14)	All	213	100	2.3	(2.0,2.6)	254	100	2.5	(2.2,2.8)	4,338	100	-0.1	(-0.5,0.3)	0.652
	MC	176	83	1.9	(1.6,2.2)	223	88	2.2	(1.9,2.5)	3,607	83	-0.3	(-0.7,0.1)	0.178
	RC	11	5	0.1	(0.0,0.2)	27	11	0.3	(0.2,0.4)	555	13	3.6*	(2.1,5.2)	< 0.001
	Other	26	12	0.3	(0.2,0.4)	4	2	0.1	(0.0,0.1)	176	4	-6.5*	(-8.7,-4.3)	< 0.001
Teenagers & Young Adults (15-24)	All	113	100	1.8	(1.5,2.1)	124	100	1.9	(1.5,2.2)	2,408	100	0.1	(-0.5,0.7)	0.825
	MC	85	75	1.3	(1.1,1.6)	114	92	1.7	(1.4,2.0)	2,091	87	0.2	(-0.3,0.8)	0.417
	RC	2	2	0.0	(0.0,0.1)	9	7	0.1	(0.0,0.2)	178	7	2.3	(-0.4,5.1)	0.094
	Other	26	23	0.4	(0.3,0.6)	1	1	0.0	(0.0,0.1)	139	6	-6.0*	(-8.8,-3.1)	< 0.001
Adults (25-64)	All	1,658	100	7.3	(6.9,7.6)	1,887	100	6.7	(6.4,7.0)	43,649	100	-0.3	(-1.4,0.7)	0.53
	MC	1,238	75	5.4	(5.1,5.7)	1,699	90	6.0	(5.7,6.3)	37,453	86	0.4	(-0.2,1.0)	0.153
	RC	105	6	0.5	(0.4,0.6)	175	9	0.6	(0.5,0.7)	3,467	8	1.4*	(0.5,2.4)	0.005
	Other	315	19	1.4	(1.3,1.6)	13	1	0.1	(0.0,0.1)	2,729	6	-9.0*	(-10.1,-7.9)	< 0.001
Older People (65-84)	All	1,125	100	16.4	(15.4,17.4)	1,928	100	22.3	(21.3,23.3)	37,721	100	1.4*	(0.8,2.0)	< 0.001
	MC	477	42	6.9	(6.3,7.5)	1,075	56	12.4	(11.6,13.1)	19,406	51	2.5*	(1.9,3.2)	< 0.001
	RC	215	19	3.1	(2.7,3.6)	791	41	9.2	(8.5,9.8)	12,222	32	4.0*	(3.0,5.1)	< 0.001
	Other	433	38	6.4	(5.8,7.0)	62	3	0.7	(0.5,0.9)	6,093	16	-9.5*	(-12.8,-6.1)	< 0.001
Elderly (85+)	All	54	100	6.2	(4.5,7.8)	281	100	20.7	(18.3,23.1)	4,134	100	5.3*	(3.9,6.7)	< 0.001
	MC	2	4	0.3	(-0.1,0.7)	13	5	1.0	(0.4,1.5)	203	5	-0.5	(-2.7,1.7)	0.636
	RC	17	31	1.9	(1.0,2.8)	250	89	18.4	(16.1,20.7)	2,802	68	9.5*	(7.6,11.5)	< 0.001
	Other	35	65	4.0	(2.7,5.4)	18	6	1.3	(0.7,2.0)	1,129	27	-4.5*	(-8.2,-0.6)	0.025
**Malignant behavior - Spinal Cord & Other CNS**														
Pediatric (0-14)	All	34	100	0.4	(0.2,0.5)	40	100	0.4	(0.3,0.5)	700	100	0.8	(-0.3,1.9)	0.132
	MC	27	79	0.3	(0.2,0.4)	24	60	0.2	(0.1,0.3)	340	49	-1.3	(-3.0,0.3)	0.109
	RC	3	9	0.0	(0.0,0.1)	14	35	0.1	(0.1,0.2)	305	44	4.5*	(0.5,8.6)	0.027
	Other	4	12	0.1	(0.0,0.1)	2	5	0.1	(0.0,0.1)	55	8	-2.6	(-5.8,0.8)	0.123
Teenagers & Young Adults (15-24)	All	9	100	0.1	(0.0,0.2)	11	100	0.2	(0.1,0.3)	171	100	0.0	(-2.1,2.1)	0.971
	MC	7	78	0.1	(0.0,0.2)	9	82	0.1	(0.0,0.2)	141	82	-0.3	(-2.6,2.0)	0.773
	RC	–	–	–	–	1	9	0.0	(0.0,0.1)	20	12	0.3	(-2.7,3.4)	0.834
	Other	2	22	0.1	(0.0,0.2)	1	9	0.0	(0.0,0.1)	10	6	-2.9*	(-5.4,-0.4)	0.031
Adults (25-64)	All	76	100	0.3	(0.2,0.4)	65	100	0.2	(0.2,0.3)	1,379	100	0.2	(-2.4,2.8)	0.894
	MC	61	80	0.2	(0.2,0.3)	52	80	0.2	(0.1,0.2)	1,179	86	0.0	(-2.7,2.8)	0.988
	RC	2	3	0.0	(0.0,0.1)	9	14	0.1	(0.0,0.1)	89	6	2.3*	(0.6,4.0)	0.009
	Other	13	17	0.1	(0.0,0.1)	4	6	0.0	(0.0,0.1)	111	8	-2.3*	(-4.0,-0.7)	0.008
Older People (65-84)	All	35	100	0.5	(0.3,0.7)	16	100	0.2	(0.1,0.3)	496	100	0.1	(-1.0,1.3)	0.795
	MC	19	54	0.3	(0.2,0.5)	11	69	0.1	(0.1,0.2)	272	55	0.7	(-0.9,2.2)	0.385
	RC	9	26	0.1	(0.0,0.2)	5	31	0.1	(0.0,0.2)	121	24	1.9	(-0.4,4.2)	0.1
	Other	7	20	0.1	(0.0,0.2)	–	–	–	–	103	21	-3.2*	(-5.1,-1.2)	0.003
Elderly (85+)	All	–	–	–	–	1	100	0.1	(-0.1,0.3)	49	100	-4.1	(-10.1,2.4)	0.213
	MC	–	–	–	–	–	–	–	–	16	33	-0.7	(-5.4,4.2)	0.744
	RC	–	–	–	–	1	100	0.1	(-0.1,0.3)	17	35	-2.6	(-9.4,4.6)	0.414
	Other	–	–	–	–	–	–	–	–	16	33	-3.2*	(-5.5,-0.9)	0.012
**Malignant behavior - Endocrine CNS**														
Pediatric (0-14)	All	7	100	0.2	(0.1,0.4)	10	100	0.2	(0.1,0.2)	272	100	0.3	(-1.0,1.7)	0.612
	MC	7	100	0.2	(0.1,0.4)	10	100	0.2	(0.1,0.2)	240	88	-0.1	(-1.7,1.5)	0.915
	RC	–	–	–	–	–	–	–	–	19	7	2.8	(-1.0,6.7)	0.132
	Other	–	–	–	–	–	–	–	–	13	5	1.8	(-1.5,5.2)	0.25
Teenagers & Young Adults (15-24)	All	12	100	0.2	(0.1,0.3)	11	100	0.2	(0.1,0.3)	253	100	0.6	(-1.2,2.5)	0.489
	MC	6	50	0.1	(0.0,0.2)	8	73	0.1	(0.0,0.2)	209	83	0.7	(-1.4,2.9)	0.482
	RC	1	8	0.0	(0.0,0.1)	3	27	0.0	(0.0,0.1)	21	8	0.8	(-1.9,3.5)	0.536
	Other	5	42	0.1	(0.0,0.2)	–	–	–	–	23	9	-1.0	(-2.8,0.8)	0.241
Adults (25-64)	All	64	100	0.3	(0.2,0.3)	17	100	0.1	(0.0,0.1)	402	100	-0.3	(-1.4,0.8)	0.585
	MC	46	72	0.2	(0.1,0.3)	12	71	0.0	(0.0,0.1)	280	70	-0.2	(-1.2,0.9)	0.765
	RC	4	6	0.0	(0.0,0.1)	3	18	0.0	(0.0,0.1)	50	12	0.4	(-1.6,2.4)	0.699
	Other	14	22	0.1	(0.0,0.2)	2	12	0.0	(0.0,0.1)	72	18	-1.9*	(-3.7,-0.1)	0.043
Older People (65-84)	All	27	100	0.4	(0.2,0.5)	10	100	0.1	(0.0,0.2)	261	100	-1.4	(-3.1,0.4)	0.115
	MC	16	59	0.4	(0.2,0.6)	7	70	0.1	(0.0,0.2)	111	43	0.9	(-1.6,3.4)	0.473
	RC	3	11	0.1	(0.0,0.1)	2	20	0.0	(0.0,0.1)	59	23	-0.1	(-1.9,1.6)	0.865
	Other	8	30	0.1	(0.0,0.2)	1	10	0.1	(-0.1,0.2)	91	35	-0.5	(-2.6,1.7)	0.637
Elderly (85+)	All	2	100	0.3	(-0.1,0.7)	–	–	–	–	61	100	0.0	(-3.0,3.0)	0.975
	MC	–	–	–	–	–	–	–	–	7	11	-1.9*	(-3.8,0.0)	0.05
	RC	2	100	0.3	(-0.1,0.7)	–	–	–	–	23	38	-1.9	(-4.0,0.3)	0.082
	Other	–	–	–	–	–	–	–	–	31	51	-2.7	(-6.0,0.8)	0.123

Abbreviations: MC, microscopically confirmed; RC, radiographically confirmed; CNS, central nervous system.
[a]Anatomical location based on the ICD-10 topography codes.
*Indicates a statistically significant departure (p<0.05) from a slope of 0.

Supplementary Table 5c. Age-Sex-Standardized Incidence Rates (ASR) per 100,000 for calendar years 1993 and 2017 separately and Average Annual Percentage Change (AAPC) for 188,340 individuals diagnosed with a primary CNS tumor according to behavior, anatomical location[a] and method of diagnosis – England, 1993-2017 - Benign tumors only

Characteristic	Method of Diagnosis	1993 only				2017 only				1993-2017				
		n	%	ASR	95% CI	n	%	ASR	95% CI	n	%	AAPC	95% CI	P-Value
**Benign behavior - Meninges**														
Pediatric (0-14)	All	2	100	0.1	(0.0,0.2)	2	100	0.1	(0.0,0.2)	83	100	-0.7	(-2.9,1.6)	0.534
	MC	1	50	0.0	(0.0,0.1)	1	50	0.0	(0.0,0.1)	69	83	-1.7	(-4.1,0.7)	0.16
	RC	–	–	–	–	1	50	0.0	(0.0,0.1)	9	11	-0.4	(-1.2,0.4)	0.264
	Other	1	50	0.0	(0.0,0.1)	–	–	–	–	5	6	-0.1	(-0.4,0.2)	0.446
Teenagers & Young Adults (15-24)	All	12	100	0.2	(0.1,0.3)	13	100	0.2	(0.1,0.3)	271	100	0.5	(-1.0,2.1)	0.479
	MC	11	92	0.2	(0.1,0.3)	10	77	0.1	(0.1,0.2)	230	85	-0.5	(-2.2,1.3)	0.597
	RC	–	–	–	–	3	23	0.0	(0.0,0.1)	30	11	1.2	(-3.1,5.7)	0.568
	Other	1	8	0.0	(0.0,0.1)	–	–	–	–	11	4	-0.4	(-5.7,5.3)	0.878
Adults (25-64)	All	383	100	1.7	(1.5,1.9)	1,148	100	4.1	(3.9,4.3)	17,849	100	3.0*	(0.8,5.3)	0.006
	MC	327	85	1.5	(1.3,1.6)	745	65	2.7	(2.5,2.8)	14,583	82	2.1*	(0.1,4.1)	0.039
	RC	11	3	0.1	(0.0,0.2)	389	34	1.4	(1.3,1.5)	2,585	14	9.9*	(7.7,12.1)	< 0.001
	Other	45	12	0.2	(0.1,0.3)	14	1	0.1	(0.0,0.1)	681	4	-5.7	(-13.0,2.3)	0.16
Older People (65-84)	All	329	100	4.8	(4.3,5.3)	1,053	100	12.2	(11.5,12.9)	15,425	100	3.0*	(0.8,5.3)	0.006
	MC	217	66	3.2	(2.7,3.6)	421	40	4.9	(4.4,5.3)	7,925	51	1.5*	(1.0,2.0)	< 0.001
	RC	29	9	0.4	(0.3,0.6)	598	57	6.9	(6.4,7.5)	5,609	36	9.0*	(6.5,11.5)	< 0.001
	Other	83	25	1.2	(0.9,1.5)	34	3	0.4	(0.3,0.5)	1,891	12	-5.3*	(-9.7,-0.7)	0.025
Elderly (85+)	All	45	100	5.6	(3.9,7.2)	251	100	18.6	(16.3,20.9)	4,178	100	4.8*	(3.4,6.2)	< 0.001
	MC	12	27	1.5	(0.6,2.3)	15	6	1.1	(0.5,1.7)	387	9	0.3	(-1.2,1.8)	0.728
	RC	7	16	0.8	(0.2,1.4)	218	87	16.1	(14.0,18.2)	2,703	65	9.3*	(7.7,10.8)	< 0.001
	Other	26	58	3.3	(2.0,4.6)	18	7	1.4	(0.7,2.0)	1,088	26	-4.6	(-9.1,0.1)	0.057
**Benign behavior - Brain**														
Pediatric (0-14)	All	9	100	0.1	(0.0,0.2)	15	100	0.2	(0.1,0.2)	443	100	1.6	(-0.7,4.0)	0.164
	MC	8	89	0.1	(0.0,0.1)	11	73	0.1	(0.0,0.2)	382	86	1.1	(-1.3,3.6)	0.345
	RC	–	–	–	–	4	27	0.1	(0.0,0.1)	41	9	0.6	(-2.9,4.3)	0.707
	Other	1	11	0.0	(0.0,0.1)	–	–	–	–	20	5	-1.0	(-3.0,1.1)	0.308
Teenagers & Young Adults (15-24)	All	4	100	0.1	(0.0,0.2)	17	100	0.3	(0.1,0.4)	276	100	4.0*	(2.2,5.8)	< 0.001
	MC	3	75	0.1	(0.0,0.2)	10	59	0.1	(0.1,0.2)	214	78	3.0*	(0.8,5.2)	0.009
	RC	–	–	–	–	7	41	0.1	(0.0,0.2)	40	14	2.4	(-2.1,7.2)	0.275
	Other	1	25	0.0	(0.0,0.1)	–	–	–	–	22	8	-0.2	(-2.7,2.4)	0.869
Adults (25-64)	All	35	100	0.1	(0.1,0.2)	116	100	0.4	(0.3,0.5)	1,411	100	5.5*	(3.8,7.3)	< 0.001
	MC	31	89	0.1	(0.1,0.2)	68	59	0.2	(0.2,0.3)	1,053	75	4.0*	(1.9,6.1)	0.001
	RC	–	–	–	–	44	38	0.2	(0.1,0.2)	240	17	6.2*	(0.4,12.3)	0.037
	Other	4	11	0.0	(0.0,0.1)	4	3	0.0	(0.0,0.1)	118	8	-0.6	(-1.9,0.7)	0.38
Older People (65-84)	All	22	100	0.3	(0.2,0.4)	34	100	0.4	(0.3,0.5)	511	100	3.5*	(1.5,5.6)	0.002
	MC	11	50	0.2	(0.1,0.3)	7	21	0.1	(0.0,0.1)	187	37	-4.3	(-17.8,11.4)	0.573
	RC	–	–	–	–	27	79	0.3	(0.2,0.4)	192	38	6.6*	(4.3,9.0)	< 0.001
	Other	11	50	0.2	(0.1,0.2)	–	–	–	–	132	26	-3.2*	(-5.6,-0.7)	0.015
Elderly (85+)	All	2	100	0.3	(-0.1,0.7)	5	100	0.4	(0.0,0.7)	95	100	1.3	(-0.8,3.4)	0.217
	MC	1	50	0.2	(-0.2,0.5)	–	–	–	–	6	6	-1.8*	(-2.5,-1.2)	0.001
	RC	–	–	–	–	4	80	0.5	(0.0,0.9)	46	48	6.3	(-6.5,20.8)	0.352
	Other	1	50	0.4	(-0.4,1.2)	1	20	0.2	(-0.2,0.6)	43	45	-2.0	(-4.6,0.8)	0.148
**Benign behavior - Spinal Cord & Other CNS**														
Pediatric (0-14)	All	–	–	–	–	16	100	0.2	(0.1,0.2)	136	100	4.1*	(1.6,6.8)	0.003
	MC	–	–	–	–	11	69	0.1	(0.0,0.2)	107	79	3.4*	(1.1,5.8)	0.006
	RC	–	–	–	–	4	25	0.1	(0.0,0.1)	15	11	1.3	(-3.5,6.4)	0.548
	Other	–	–	–	–	1	6	0.0	(0.0,0.1)	14	10	0.7	(-0.9,2.4)	0.35
Teenagers & Young Adults (15-24)	All	10	100	0.3	(0.1,0.5)	25	100	0.4	(0.2,0.5)	409	100	0.5	(-1.0,2.0)	0.51
	MC	9	90	0.3	(0.1,0.4)	17	68	0.3	(0.1,0.4)	353	86	-0.3	(-1.8,1.2)	0.658
	RC	–	–	–	–	8	32	0.1	(0.0,0.2)	40	10	4.9	(-0.1,10.2)	0.056
	Other	1	10	0.0	(0.0,0.1)	–	–	–	–	16	4	-2.4	(-10.3,6.2)	0.57
Adults (25-64)	All	196	100	0.9	(0.7,1.0)	503	100	1.8	(1.6,1.9)	8,848	100	3.2*	(1.5,5.0)	< 0.001
	MC	163	83	0.7	(0.6,0.8)	309	61	1.1	(1.0,1.2)	6,784	77	1.6	(-2.1,5.4)	0.41
	RC	7	4	0.0	(0.0,0.1)	187	37	0.7	(0.6,0.8)	1,497	17	11.0*	(7.7,14.3)	< 0.001
	Other	26	13	0.1	(0.1,0.2)	7	1	0.0	(0.0,0.1)	567	6	-2.8	(-18.8,16.3)	0.754
Older People (65-84)	All	65	100	0.9	(0.7,1.2)	222	100	2.6	(2.2,2.9)	3,079	100	3.3*	(1.7,4.8)	< 0.001
	MC	45	69	0.7	(0.5,0.8)	83	37	1.0	(0.8,1.2)	1,721	56	0.1	(-0.7,1.0)	0.735
	RC	3	5	0.1	(0.0,0.2)	135	61	1.6	(1.3,1.8)	1,000	32	11.6*	(9.4,14.0)	< 0.001
	Other	17	26	0.3	(0.1,0.4)	4	2	0.1	(0.0,0.1)	358	12	-5.4	(-15.9,6.5)	0.358
Elderly (85+)	All	3	100	0.5	(-0.1,1.1)	14	100	1.0	(0.5,1.5)	188	100	2.6*	(0.7,4.6)	0.01
	MC	–	–	–	–	2	14	0.2	(-0.1,0.6)	31	16	-2.0	(-5.2,1.2)	0.204
	RC	–	–	–	–	12	86	0.9	(0.4,1.3)	100	53	3.5*	(0.4,6.6)	0.03
	Other	3	100	0.5	(-0.1,1.1)	–	–	–	–	57	30	2.4	(-1.0,5.8)	0.16
**Benign behavior - Endocrine CNS**														
Pediatric (0-14)	All	1	100	0.0	(0.0,0.1)	5	100	0.1	(0.0,0.1)	101	100	2.3	(-1.5,6.2)	0.228
	MC	–	–	–	–	3	60	0.0	(0.0,0.1)	69	68	-1.2	(-4.9,2.6)	0.502
	RC	–	–	–	–	1	20	0.0	(0.0,0.1)	25	25	4.2	(-4.2,13.3)	0.302
	Other	1	100	0.0	(0.0,0.1)	1	20	0.0	(0.0,0.1)	7	7	-0.3	(-0.9,0.2)	0.178
Teenagers & Young Adults (15-24)	All	23	100	0.4	(0.2,0.5)	42	100	0.6	(0.4,0.8)	717	100	1.1	(-0.2,2.5)	0.096
	MC	13	57	0.2	(0.1,0.3)	21	50	0.3	(0.2,0.4)	449	63	-0.6	(-2.2,1.0)	0.436
	RC	1	4	0.0	(0.0,0.1)	20	48	0.3	(0.2,0.4)	195	27	7.2*	(4.9,9.5)	< 0.001
	Other	9	39	0.1	(0.0,0.2)	1	2	0.0	(0.0,0.1)	73	10	-3.1*	(-5.9,-0.3)	0.034
Adults (25-64)	All	334	100	1.4	(1.3,1.6)	508	100	1.8	(1.6,1.9)	10,157	100	0.5	(-3.7,4.8)	0.829
	MC	275	82	1.2	(1.0,1.3)	352	69	1.2	(1.1,1.4)	8,022	79	0.4	(-0.2,1.1)	0.182
	RC	14	4	0.1	(0.0,0.1)	147	29	0.5	(0.4,0.6)	1,515	15	7.4*	(1.4,13.7)	0.014
	Other	45	13	0.2	(0.1,0.2)	9	2	0.0	(0.0,0.1)	620	6	-2.2	(-4.7,0.4)	0.094
Older People (65-84)	All	123	100	1.8	(1.5,2.1)	253	100	2.9	(2.6,3.3)	4,758	100	2.2	(-2.3,6.9)	0.346
	MC	81	66	1.2	(0.9,1.4)	159	63	1.8	(1.5,2.1)	3,355	71	2.1	(-2.1,6.5)	0.331
	RC	11	9	0.2	(0.1,0.3)	90	36	1.0	(0.8,1.3)	978	21	8.3*	(7.1,9.5)	< 0.001
	Other	31	25	0.5	(0.3,0.6)	4	2	0.1	(0.0,0.1)	425	9	-6.8	(-13.5,0.4)	0.063
Elderly (85+)	All	6	100	0.8	(0.1,1.4)	42	100	3.1	(2.1,4.0)	423	100	6.5*	(4.8,8.2)	< 0.001
	MC	4	67	0.5	(0.0,1.0)	5	12	0.6	(0.1,1.1)	106	25	1.1	(-1.1,3.3)	0.33
	RC	–	–	–	–	35	83	2.6	(1.7,3.4)	216	51	9.6*	(7.3,12.0)	< 0.001
	Other	2	33	0.3	(-0.1,0.7)	2	5	0.2	(-0.1,0.4)	101	24	-5.0	(-11.8,2.3)	0.172

Abbreviations: MC, microscopically confirmed; RC, radiographically confirmed; CNS, central nervous system.
[a]Anatomical location based on the ICD-10 topography codes.
*Indicates a statistically significant departure (p<0.05) from a slope of 0.

Supplementary Table 5d. Age-Sex-Standardized Incidence Rates (ASR) per 100,000 for calendar years 1993 and 2017 separately and Average Annual Percentage Change (AAPC) for 188,340 individuals diagnosed with a primary CNS tumor according to behavior, anatomical location[a] and method of diagnosis – England, 1993-2017 - Uncertain tumors only

Characteristic	Method of Diagnosis	1993 only				2017 only				1993-2017				
		n	%	ASR	95% CI	n	%	ASR	95% CI	n	%	AAPC	95% CI	P-Value
**Uncertain behavior - Meninges**														
Pediatric (0-14)	All	–	–	–	–	1	100	0.0	(0.0,0.1)	28	100	4.0*	(1.4,6.8)	0.005
	MC	–	–	–	–	1	100	0.0	(0.0,0.1)	25	89	3.9*	(0.9,7.0)	0.014
	RC	–	–	–	–	–	–	–	–	1	4	–	–	–
	Other	–	–	–	–	–	–	–	–	2	7	2.5	–	–
Teenagers & Young Adults (15-24)	All	–	–	–	–	3	100	0.0	(0.0,0.1)	40	100	2.6	(-0.9,6.2)	0.139
	MC	–	–	–	–	3	100	0.0	(0.0,0.1)	39	98	2.5	(-1.4,6.5)	0.189
	RC	–	–	–	–	–	–	–	–	0	0	–	–	–
	Other	–	–	–	–	–	–	–	–	1	3	–	–	–
Adults (25-64)	All	3	100	0.0	(0.0,0.1)	153	100	0.5	(0.5,0.6)	1,476	100	12.2*	(10.1,14.4)	< 0.001
	MC	2	67	0.0	(0.0,0.1)	147	96	0.5	(0.4,0.6)	1,364	92	13.0*	(10.2,15.9)	< 0.001
	RC	1	33	0.0	(0.0,0.1)	6	4	0.1	(0.0,0.1)	66	4	3.4*	(1.7,5.0)	0.001
	Other	–	–	–	–	–	–	–	–	46	3	-1.1	(-2.9,0.7)	0.2
Older People (65-84)	All	1	100	0.0	(0.0,0.1)	93	100	1.1	(0.9,1.3)	1,035	100	11.0*	(7.9,14.1)	< 0.001
	MC	1	100	0.0	(0.0,0.1)	81	87	0.9	(0.7,1.1)	830	80	15.0*	(11.9,18.3)	< 0.001
	RC	–	–	–	–	9	10	0.1	(0.0,0.2)	127	12	4.5*	(2.2,6.9)	< 0.001
	Other	–	–	–	–	3	3	0.0	(0.0,0.1)	78	8	-0.7	(-2.9,1.6)	0.531
Elderly (85+)	All	–	–	–	–	9	100	0.7	(0.2,1.1)	121	100	5.1*	(1.9,8.3)	0.003
	MC	–	–	–	–	2	22	0.2	(-0.1,0.6)	23	19	7.4*	(0.7,14.5)	0.033
	RC	–	–	–	–	7	78	0.5	(0.1,0.9)	56	46	1.6	(-2.5,5.9)	0.423
	Other	–	–	–	–	–	–	–	–	42	35	2.0	(-1.8,5.9)	0.289
**Uncertain behavior - Brain**														
Pediatric (0-14)	All	14	100	0.2	(0.1,0.2)	42	100	0.4	(0.3,0.5)	2,278	100	2.5*	(1.8,3.3)	< 0.001
	MC	6	43	0.1	(0.0,0.1)	31	74	0.3	(0.2,0.4)	2,082	91	4.5*	(1.7,7.5)	0.002
	RC	1	7	0.0	(0.0,0.1)	10	24	0.1	(0.0,0.2)	130	6	3.1*	(1.1,5.1)	0.004
	Other	7	50	0.1	(0.0,0.1)	1	2	0.0	(0.0,0.1)	66	3	-2.5*	(-4.4,-0.6)	0.015
Teenagers & Young Adults (15-24)	All	4	100	0.1	(0.0,0.1)	26	100	0.4	(0.2,0.5)	932	100	3.9*	(2.7,5.2)	< 0.001
	MC	3	75	0.0	(0.0,0.1)	23	88	0.3	(0.2,0.5)	823	88	4.1*	(2.8,5.3)	< 0.001
	RC	–	–	–	–	3	12	0.0	(0.0,0.1)	66	7	3.7*	(0.2,7.2)	0.039
	Other	1	25	0.0	(0.0,0.1)	–	–	–	–	43	5	-3.5*	(-6.3,-0.6)	0.021
Adults (25-64)	All	73	100	0.3	(0.2,0.4)	143	100	0.5	(0.4,0.6)	3,872	100	1.6*	(0.9,2.2)	< 0.001
	MC	23	32	0.1	(0.1,0.1)	96	67	0.3	(0.3,0.4)	2,471	64	5.3*	(2.4,8.3)	< 0.001
	RC	10	14	0.1	(0.0,0.1)	42	29	0.1	(0.1,0.2)	694	18	1.5	(-4.1,7.4)	0.615
	Other	40	55	0.2	(0.1,0.2)	5	3	0.0	(0.0,0.1)	707	18	-5.0*	(-6.2,-3.8)	< 0.001
Older People (65-84)	All	180	100	2.6	(2.3,3.0)	108	100	1.2	(1.0,1.5)	4,822	100	-3.8*	(-5.5,-2.1)	< 0.001
	MC	11	6	0.2	(0.1,0.3)	21	19	0.3	(0.2,0.4)	599	12	2.7*	(0.3,5.2)	0.028
	RC	43	24	0.6	(0.4,0.8)	74	69	0.8	(0.7,1.0)	2,052	43	0.4	(-1.5,2.3)	0.698
	Other	126	70	1.8	(1.5,2.2)	13	12	0.2	(0.1,0.2)	2,171	45	-11.4*	(-15.3,-7.3)	< 0.001
Elderly (85+)	All	25	100	2.9	(1.7,4.1)	37	100	2.8	(1.9,3.7)	1,502	100	-1.3	(-4.1,1.6)	0.37
	MC	2	8	0.3	(-0.1,0.7)	1	3	0.1	(-0.1,0.3)	43	3	-2.9	(-6.6,0.9)	0.12
	RC	9	36	1.0	(0.3,1.7)	33	89	2.5	(1.6,3.3)	722	48	4.2*	(1.9,6.5)	< 0.001
	Other	14	56	1.6	(0.8,2.5)	3	8	0.2	(0.0,0.5)	737	49	-9.5*	(-14.9,-3.8)	0.001
**Uncertain behavior - Spinal Cord & Other CNS**														
Pediatric (0-14)	All	9	100	0.1	(0.0,0.2)	12	100	0.1	(0.1,0.2)	322	100	1.4	(-0.6,3.3)	0.157
	MC	6	67	0.1	(0.0,0.1)	6	50	0.1	(0.0,0.1)	255	79	1.1	(-0.6,2.7)	0.194
	RC	–	–	–	–	5	42	0.0	(0.0,0.1)	50	16	3.0*	(0.8,5.3)	0.008
	Other	3	33	0.1	(0.0,0.2)	1	8	0.0	(0.0,0.1)	17	5	-5.4*	(-6.3,-4.4)	< 0.001
Teenagers & Young Adults (15-24)	All	2	100	0.1	(0.0,0.1)	7	100	0.1	(0.0,0.2)	158	100	3.6*	(1.7,5.6)	0.001
	MC	1	50	0.0	(0.0,0.1)	6	86	0.1	(0.0,0.2)	141	89	4.2*	(2.1,6.3)	< 0.001
	RC	–	–	–	–	1	14	0.0	(0.0,0.1)	9	6	-0.1	(-0.7,0.4)	0.566
	Other	1	50	0.0	(0.0,0.1)	–	–	–	–	8	5	-0.6	(-1.4,0.3)	0.158
Adults (25-64)	All	26	100	0.1	(0.1,0.1)	69	100	0.2	(0.2,0.3)	1,019	100	4.1	(-0.1,8.4)	0.055
	MC	17	65	0.1	(0.0,0.1)	55	80	0.2	(0.1,0.2)	836	82	3.8*	(0.2,7.6)	0.039
	RC	2	8	0.1	(0.0,0.1)	13	19	0.1	(0.0,0.1)	93	9	2.5	(-2.1,7.4)	0.295
	Other	7	27	0.0	(0.0,0.1)	1	1	0.0	(0.0,0.1)	90	9	0.9	(-1.5,3.3)	0.447
Older People (65-84)	All	17	100	0.2	(0.1,0.4)	27	100	0.3	(0.2,0.4)	338	100	2.7	(-0.2,5.7)	0.068
	MC	2	12	0.1	(0.0,0.1)	15	56	0.2	(0.1,0.3)	138	41	3.5*	(0.5,6.7)	0.022
	RC	4	24	0.1	(0.0,0.2)	12	44	0.1	(0.1,0.2)	93	28	3.5*	(1.0,6.0)	0.008
	Other	11	65	0.2	(0.1,0.3)	–	–	–	–	107	32	-3.8*	(-5.3,-2.2)	< 0.001
Elderly (85+)	All	3	100	0.4	(-0.1,0.8)	1	100	0.1	(-0.1,0.3)	37	100	-1.4	(-4.0,1.3)	0.291
	MC	–	–	–	–	–	–	–	–	4	11	-6.0	(-54.8,95.5)	0.751
	RC	2	67	0.4	(-0.1,0.8)	1	100	0.1	(-0.1,0.3)	17	46	-2.0	(-4.6,0.7)	0.128
	Other	1	33	0.4	(-0.4,1.2)	–	–	–	–	16	43	-2.5	(-6.7,2.0)	0.233
**Uncertain behavior - Endocrine CNS**														
Pediatric (0-14)	All	23	100	0.3	(0.2,0.4)	24	100	0.2	(0.1,0.3)	466	100	-0.8	(-1.7,0.2)	0.122
	MC	18	78	0.2	(0.1,0.3)	19	79	0.2	(0.1,0.3)	391	84	-1.1*	(-2.2,-0.1)	0.038
	RC	3	13	0.0	(0.0,0.1)	3	13	0.1	(0.0,0.2)	46	10	2.1	(-0.3,4.5)	0.086
	Other	2	9	0.1	(0.0,0.2)	2	8	0.1	(0.0,0.1)	29	6	-0.5	(-3.1,2.1)	0.66
Teenagers & Young Adults (15-24)	All	3	100	0.1	(0.0,0.2)	18	100	0.3	(0.1,0.4)	267	100	0.6	(-0.8,2.0)	0.385
	MC	2	67	0.1	(0.0,0.1)	13	72	0.2	(0.1,0.3)	180	67	0.5	(-1.2,2.3)	0.536
	RC	–	–	–	–	4	22	0.1	(0.0,0.2)	41	15	4.9*	(1.8,8.1)	0.004
	Other	1	33	0.0	(0.0,0.1)	1	6	0.0	(0.0,0.1)	46	17	-3.9*	(-7.1,-0.5)	0.026
Adults (25-64)	All	30	100	0.2	(0.1,0.2)	54	100	0.2	(0.1,0.2)	1,393	100	1.5	(-2.4,5.6)	0.453
	MC	18	60	0.1	(0.0,0.1)	38	70	0.1	(0.1,0.2)	932	67	0.1	(-1.0,1.2)	0.886
	RC	5	17	0.1	(0.0,0.1)	10	19	0.0	(0.0,0.1)	192	14	0.2	(-1.6,2.0)	0.843
	Other	7	23	0.1	(0.0,0.1)	6	11	0.0	(0.0,0.1)	269	19	-2.6	(-5.9,0.8)	0.133
Older People (65-84)	All	5	100	0.1	(0.0,0.3)	23	100	0.3	(0.2,0.4)	732	100	-0.5	(-2.4,1.4)	0.612
	MC	–	–	–	–	16	70	0.2	(0.1,0.3)	292	40	1.8	(-0.9,4.7)	0.193
	RC	1	20	0.0	(0.0,0.1)	4	17	0.1	(0.0,0.2)	195	27	0.5	(-1.5,2.4)	0.623
	Other	4	80	0.1	(0.0,0.2)	3	13	0.0	(0.0,0.1)	245	33	-6.1*	(-11.1,-0.8)	0.024
Elderly (85+)	All	2	100	0.3	(-0.1,0.7)	2	100	0.4	(-0.2,1.0)	113	100	0.3	(-2.9,3.5)	0.868
	MC	–	–	–	–	–	–	–	–	5	4	-0.3	(-15.9,18.2)	0.955
	RC	–	–	–	–	2	100	0.4	(-0.2,1.0)	51	45	-0.5	(-5.5,4.7)	0.835
	Other	2	100	0.3	(-0.1,0.7)	–	–	–	–	57	50	-1.4	(-4.2,1.5)	0.33

Abbreviations: MC, microscopically confirmed; RC, radiographically confirmed; CNS, central nervous system.

[a]Anatomical location based on the ICD-10 topography codes.

*Indicates a statistically significant departure (p<0.05) from a slope of 0.

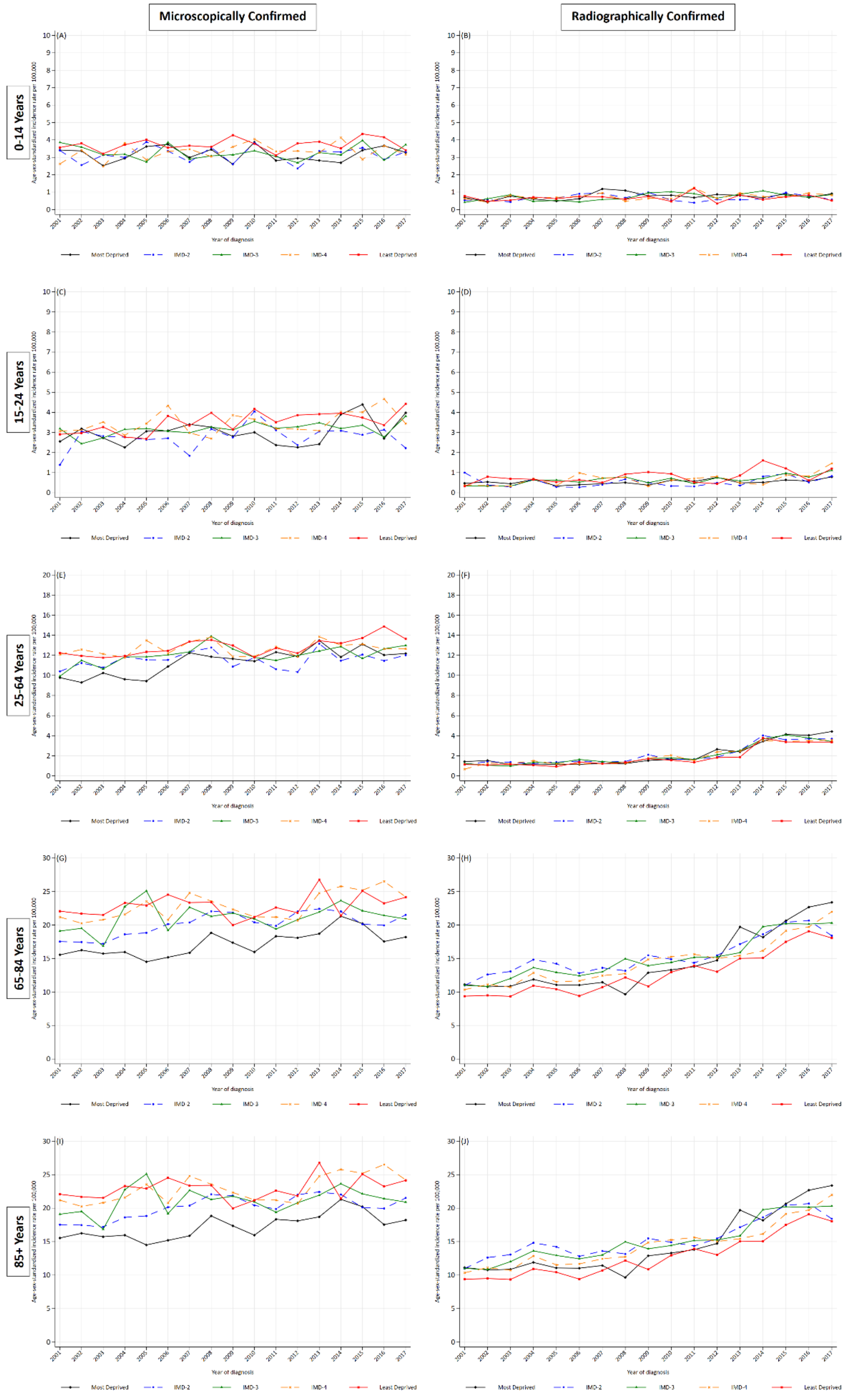

IMD - Index of Multiple Deprivation

Supplementary Figure 2. Incidence rate of primary CNS tumors by method of diagnosis, age-group and index of multiple deprivation (IMD) - England, 2001-2017

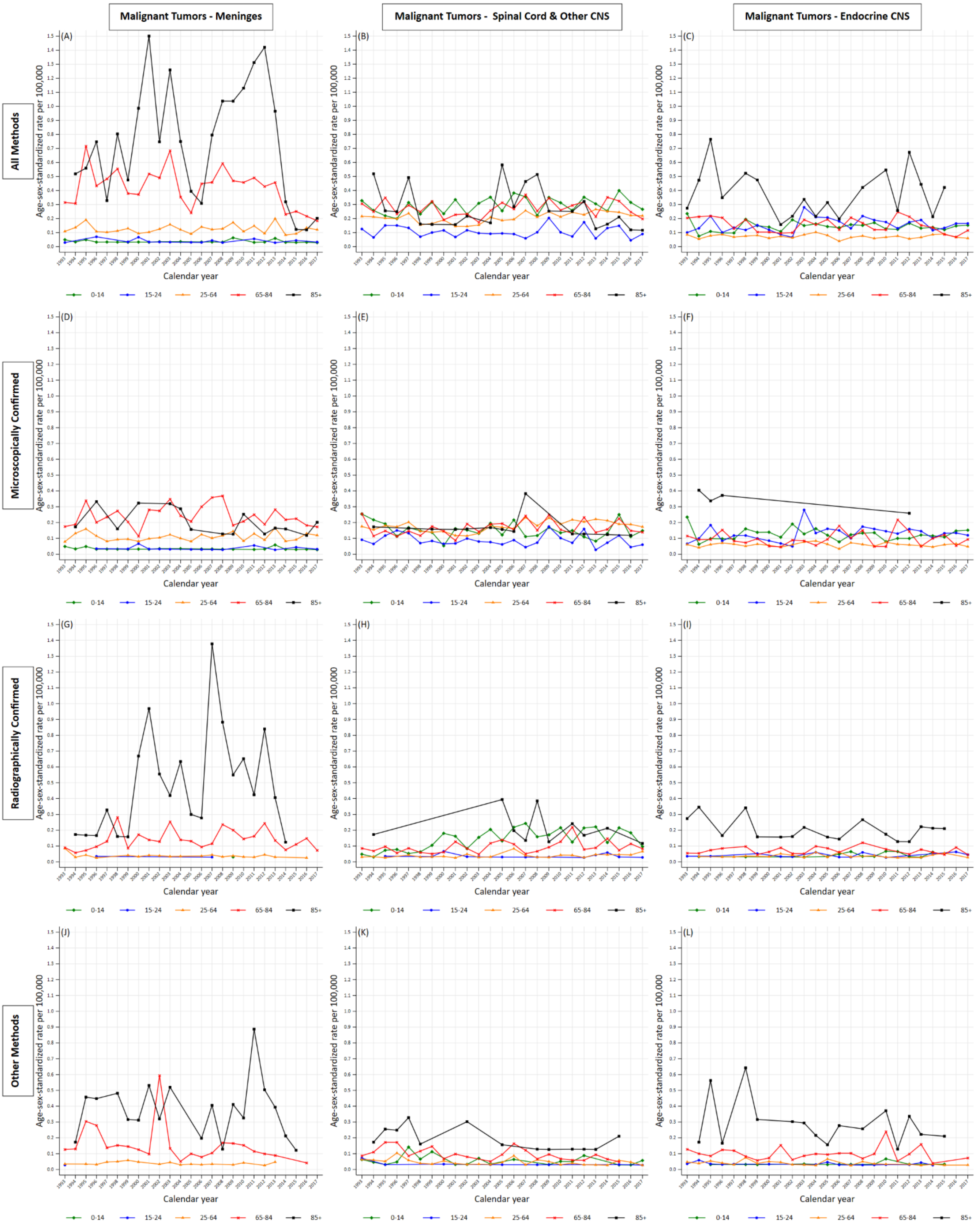

Supplementary Figure 3. Incidence of primary CNS tumors by anatomical location (malignant tumors only), method of diagnosis and age-group - England, 1993-2017

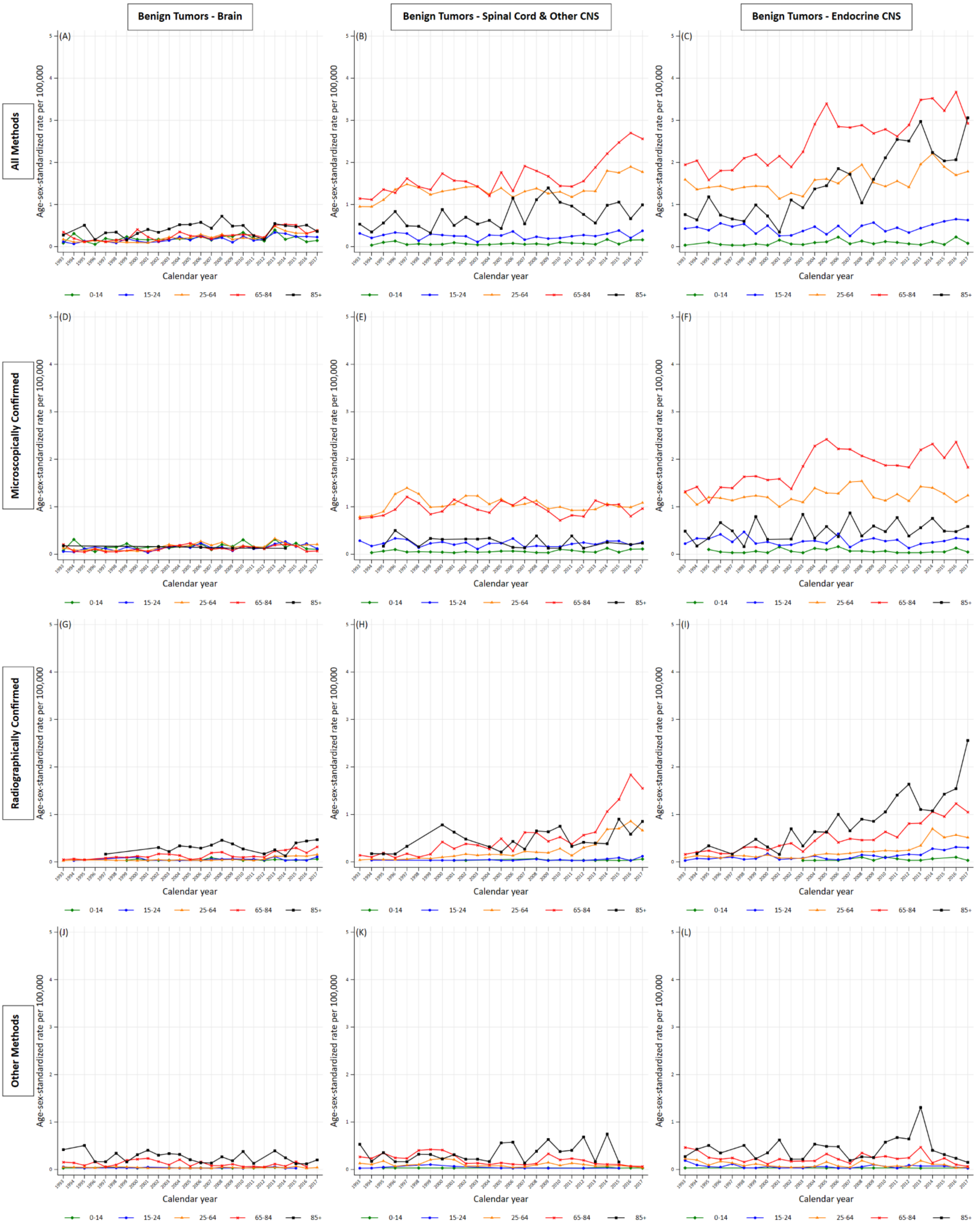

Supplementary Figure 4. Incidence of primary CNS tumors by anatomical location (benign tumors only), method of diagnosis and age-group - England, 1993-2017

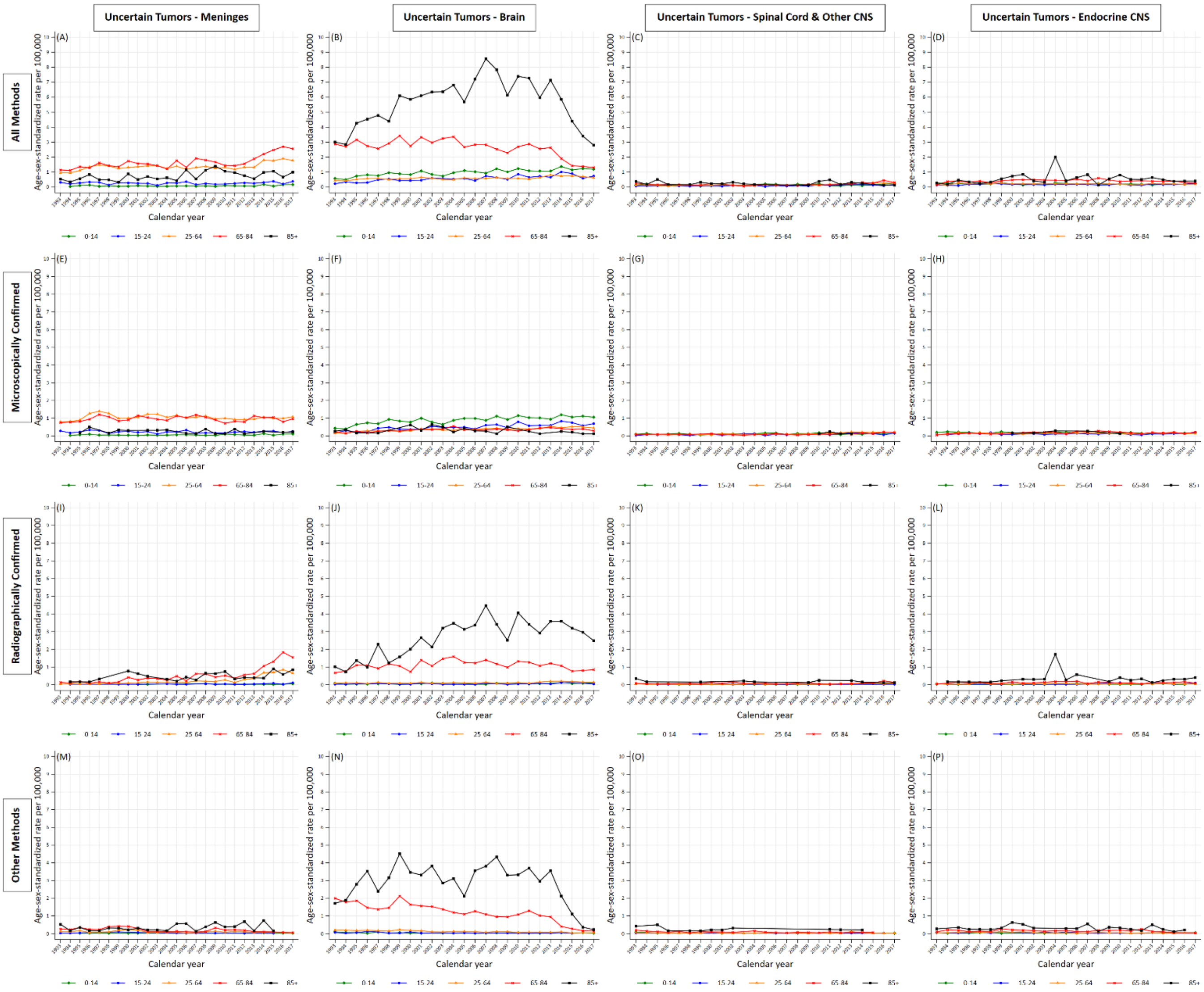

Supplementary Figure 5. Incidence of primary CNS tumors by anatomical location (uncertain tumors only), method of diagnosis and age-group - England, 1993-2017
